# Mycobacterium tuberculosis-Specific CD4 T Cells Expressing Transcription Factors T-Bet or RORγT Associate with Bacterial Control in Granulomas

**DOI:** 10.1128/mbio.00477-23

**Published:** 2023-04-11

**Authors:** Nicole L. Grant, Kristen Kelly, Pauline Maiello, Helena Abbott, Shelby O’Connor, Philana Ling Lin, Charles A. Scanga, JoAnne L. Flynn

**Affiliations:** a Department of Infectious Disease and Microbiology, University of Pittsburgh Graduate School of Public Health, Pittsburgh, Pennsylvania, USA; b Department of Microbiology and Molecular Genetics, University of Pittsburgh School of Medicine, Pittsburgh, Pennsylvania, USA; c Department of Pathology and Laboratory Medicine, University of Wisconsin-Madison, Madison Wisconsin, USA; d Department of Pediatrics, Children's Hospital of Pittsburgh of the University of Pittsburgh Medical Center, Pittsburgh, Pennsylvania, USA; e Center for Vaccine Research, University of Pittsburgh, Pittsburgh, Pennsylvania, USA; Washington University in St. Louis

**Keywords:** MHC class II tetramer, *Mycobacterium tuberculosis*, T cell, granuloma, lung infection, macaque, tuberculosis

## Abstract

Despite the extensive research on CD4 T cells within the context of Mycobacterium tuberculosis (Mtb) infections, few studies have focused on identifying and investigating the profile of Mtb-specific T cells within lung granulomas. To facilitate the identification of Mtb-specific CD4 T cells, we identified immunodominant epitopes for two Mtb proteins, namely, Rv1196 and Rv0125, using a Mauritian cynomolgus macaque model of Mtb infection, thereby providing data for the synthesis of MHC class II tetramers. Using tetramers, we identified Mtb-specific cells within different immune compartments, postinfection. We found that granulomas were enriched sites for Mtb-specific cells and that tetramer^+^ cells had increased frequencies of the activation marker CD69 as well as the transcription factors T-bet and RORγT, compared to tetramer negative cells within the same sample. Our data revealed that while the frequency of Rv1196 tetramer^+^ cells was positively correlated with the granuloma bacterial burden, the frequency of RORγT or T-bet within tetramer^+^ cells was inversely correlated with the granuloma bacterial burden, thereby highlighting the importance of having activated, polarized, Mtb-specific cells for the control of Mtb in lung granulomas.

## INTRODUCTION

Mycobacterium tuberculosis (Mtb), the causative agent of TB disease, remains a global health problem, despite over a century of observations and research. The bacterium itself is a complex organism with roughly 4,000 genes and an estimated 3,924 proteins, of which 204 are predicted to be secreted (1). Despite this large antigenic potential, immunodominant antigens overlap between human and macaque species (NHPs) (2). Several immunodominant antigens to Mtb have been studied, with some being incorporated into vaccines that are currently in clinical trials, although the functions and relevance to virulence for many of these proteins remain incompletely understood. The immunodominant antigens that are included in several vaccine candidates consist of the well-known secreted proteins ESAT-6 and CFP-10, as well as the serine protease Rv0125 and the functionally less-understood protein Rv1196 (3–9). CD4 T cells have been extensively studied in the context of Mtb infections, with critical functions being related to their production of proinflammatory cytokines and interactions with other cells, including CD8 T cells and macrophages (10, 11). While many studies investigating CD4 T cells in TB disease utilize Mtb peptide stimulation, flow staining using peptide:MHC complexes (i.e., tetramers) in peripheral samples, or single cell analyses, few provide insight into the presence and function of Mtb-specific CD4 T cells in lung granulomas, particularly in nonhuman primate models (12–16).

While the large antigenic repertoire of Mtb could contribute to enhanced pathogen recognition by host T cells, it may also contribute to the difficulty in its clearance and to the complexity of immune responses, as some antigens may act as “decoys” and evade host protective responses (17). Following infection, granulomas are formed through the migration of innate and adaptive cells in response to the initiating bacillus, thereby contributing to the overall microenvironment (18, 19). Previous studies investigating T cells in granulomas provide evidence that, while they make up roughly 30 to 40% of all cells, only a fraction produce proinflammatory cytokines (20). Hypotheses as to why T cells may not produce high frequencies of proinflammatory cytokines within lung granulomas include, but are not limited to: (i) T cells in lung granulomas experience constant stimulation by antigens and thus become functionally exhausted; (ii) the granuloma is comprised of spatial cellular compartments, which limit interactions between APCs and specific T cells; and (iii) nonspecific T cells are recruited to lung granulomas, and, thus, the functionality is dependent on antigen-specificity. Previous studies from our lab and others indicate that there are low levels of multiple exhaustion markers on granuloma T cells, and they have predicted how the cellular spatial environment of the granuloma contributes to macrophage:T cell interactions (17, 21–23). However, the difficulty in studying antigen specific T cells in granulomas, which often have limited cells for analysis, presents a challenge in testing these possibilities. Tools with which to identify Mtb-specific T cells within the granuloma would provide valuable insight into the phenotype and potential functionality of these cells.

Tetramers are a valuable tool for the identification and investigation of the functional attributes of antigen-specific cells; however, given the wide polymorphic diversity in MHC I and II molecules in humans and in commonly used NHP models, designing useful tetramers can be challenging (24–33). Mauritian cynomolgus macaques (MCMs) provide a useful model with which to develop and use tetramers to study antigen-specific cells, as these NHPs emerged from a small, isolated, founder population, thereby leading to a substantially reduced MHC diversity (29, 34, 35). The diversity in MHC I and II molecules in MCMs has been well-studied, and evidence of 7 distinct groups, based on the expression of major MHC haplotypes, has been provided (35). Further limiting the use of MCMs to include only those from the M1 haplotype greatly reduces the major MHC I and II allele variation (35). MCMs have been developed as a model for Mtb infection, presenting with granulomas and other pathologies that are similar to those of humans and other NHP species, with a higher susceptibility to active TB disease, similar to that of rhesus macaques (36–38).

Here, we mapped the dominant epitopes for two immunodominant Mtb proteins and acquired tetramers for these antigens. Using these new tetramers, plus two tetramers that had previously been developed to identify CD4 T cells that were specific for CFP-10, we investigated Mtb-specific T cells within infected MCMs, thereby providing insight into the presence of Mtb-specific T cells in the blood, BAL, lungs, lung granulomas, lymph nodes (LNs), and extrapulmonary sites (EP) (3). Furthermore, we investigated the function of Mtb-specific CD4 T cells in lung lesions by using transcription factors and activation markers, and we demonstrated an association between Mtb-specific CD4 T cells expressing T-bet or RORγT and a reduction in the bacterial burden within granulomas.

## RESULTS

### Identifying a dominant epitope for Rv1196 and Rv0125 in M1/M1 MCMs.

**FIG 1:**
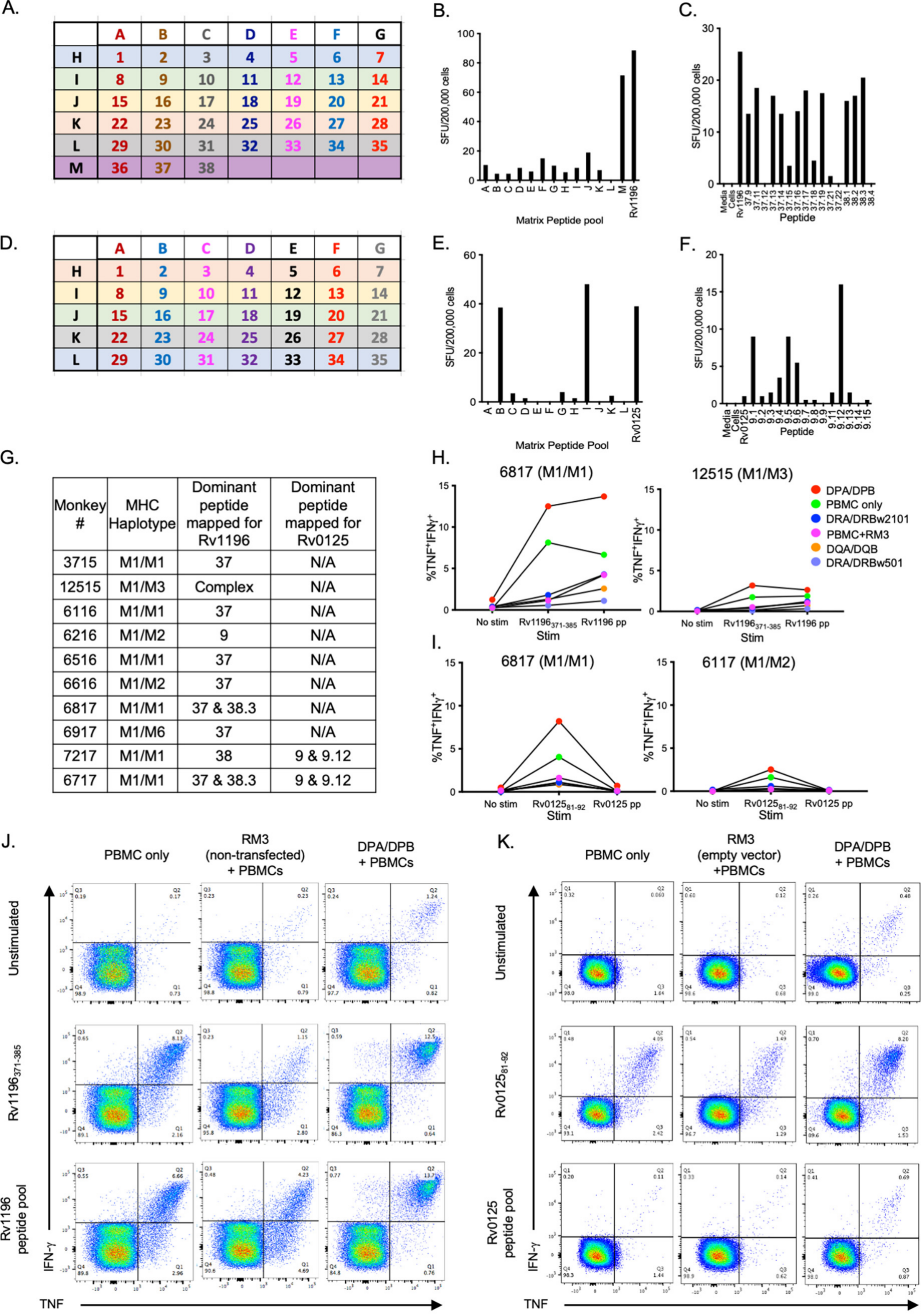
Epitope mapping and MHC allele restriction for Mtb proteins Rv1196 and Rv0125. (A) Peptide pools following a matrix mapping strategy were generated for Rv1196. The letters indicate peptide pools, and the numbers indicate individual, 20-amino acid peptides (Table S1). IFN-γ ELISPOT, using stimulated PBMCs with Rv1196 peptide pools from a matrix pooling strategy (B) as well as truncated and adjacent peptides (C). (D) A similar approach was performed for Rv0125 peptide pools, following a matrix mapping strategy for Rv0125 (Table S2). IFN-γ ELISPOT, using stimulated PBMC peptide pools from a matrix pooling strategy (E) as well as truncated and adjacent peptides (F). (G) List of Mtb-infected MCMs used for epitope mapping for Rv1196 and Rv0125. (H and I) T cells were expanded from PBMCs isolated from MCMs and cocultured for 12 h with either Rv1196_371-385_ peptide (I) or Rv0125_81-92_ peptide (J) and stimulated with RM3 cells that had been transfected with different M1/M1 MHC alleles (color legend shown). Flow cytometry was performed to determine the MHC II-presenting allele. The highest frequency of TNF^+^IFN-γ^+^ production from the CD4^+^ cells was observed when cultured with either peptide and RM3 cells that had been transfected with the DPA/DPB allele. (J and K) Representative flow cytometry plots showing expanded T cells cocultured with DPA/DPB-transfected (or untransfected) RM3 cells and unstimulated or stimulated with the Rv1196_371-385_ or Rv1196 peptide pool (J) or the Rv0125_81-92_ or Rv0125 peptide pool (K) for animal number 6817 (M1/M1).

A total of 10 Mtb-infected MCMs (including M1 homozygous and M1 heterozygous animals) were used to epitope map two Mtb proteins, namely, Rv1196 and Rv0125, using IFN-γ ELISPOTs (Fig. 1). To investigate the dominant epitope binding region for these proteins, we used PBMCs from various time points throughout infection, with PBMCs from 4 to 8 weeks p.i. eliciting the best overall IFN-γ responses. To narrow down the dominant epitope binding region, individual, 20-amino acid (a.a.) peptides with overlapping 10-a.a. regions were pooled in a matrix format (Fig. 1A and D; Tables S1 and S2).

10.1128/mbio.00477-23.6 TABLE S1 Peptides used for mapping Rv1196. Download Table S1, TIF file, 4.7 MB. Copyright © 2023 Grant et al. 2023 Grant et al. https://creativecommons.org/licenses/by/4.0/ This content is distributed under the terms of the Creative Commons Attribution 4.0 International license.

10.1128/mbio.00477-23.7 TABLE S2 Peptides used for mapping Rv0125. Download Table S2, TIF file, 4.7 MB. Copyright © 2023 Grant et al. 2023 Grant et al. https://creativecommons.org/licenses/by/4.0/ This content is distributed under the terms of the Creative Commons Attribution 4.0 International license.

For mapping Rv1196, the highest IFN-γ responses were to peptide number 37 and peptide number 38, indicating that the optimal binding region likely included the overlapping 10-a.a. region (Fig. 1B). To further evaluate the amino acids involved in binding, we used truncated and shifted peptides of 9 to 20 a.a. in length in PBMC IFN-γ ELISPOTs (Fig. 1C; Table S1). For Rv1196, we identified the optimal binding region to be between amino acids 371 and 385 (peptide Rv1196 38.3) (Fig. 1G). Only peptides containing the VM (valine-methionine, a.a. 383 and 384) elicited IFN-γ responses, suggesting that VM is critical in peptide binding to M1 MHC II molecules (Table S1).

A similar approach was taken using peptides for Rv0125, with the highest responses being elicited from Rv0125 peptide 9 (Rv0125_81-100_) and by the truncated peptide 9.12 (Rv0125_81-92_) (Fig. 1D–G; Table S2). Results from IFN-γ ELISPOTs revealed that the dominant epitope binding region for Rv1196 and Rv0125 were 15 and 12 a.a. in length, respectively. As MHC I binds peptides of smaller sizes, it is likely that these peptides are bound by MHC II and recognized by CD4 T cells; however, based on size, Rv0125_81-92_ is small enough to be bound by MHC I (39).

### Rv1196_371-385_ and Rv0125_81-92_ are presented by the DPA/DPB allele.

There are two main steps in the tetramer design process: (i) identifying the dominant epitope, as outlined above, and (ii) identifying the MHC and allele presenting the dominant peptide to either CD8 or CD4 T cells (MHC I or MHC II, respectively). This critical step involves generating T cell lines, APC lines, and cell lines expressing specific MHC alleles. The generation of T cell lines has been described in humans, mice, and NHP, using media supplemented with recombinant IL-2 and IL-7 (40, 41). Although it has been reported that T cell lines can be passaged for 10 weeks or more and still remain functional, we did not observe cytokine production in our T cell lines that were grown and stimulated for more than 3 weeks in culture with irradiated BLCLs and peptides (42). We developed a protocol for expanding T cells, based on protocols provided by the University of Wisconsin. Following a 12 h coculture experiment in which our expanded T cell lines were incubated with autologous peptide stimulated PBMCs, we performed flow cytometry staining for surface markers (CD3, CD4, and CD8) and cytokines (IFN-γ and TNF) to determine whether the peptides were presented to CD4 or CD8 T cells. This demonstrated that CD4 T cells recognized and produced IFN-γ and TNF in response to both peptides (Rv1196_371-385_ and Rv0125_81-92_) and were therefore likely MHC II-restricted (Fig. S1B).

10.1128/mbio.00477-23.1 FIG S1 T cell culture staining, plasmids, and RM3 cell transfection plots. (A) Table of plasmid reagents for the transfection of RM3 cells. (B) Expanded T cells were cocultured with peptide-pulsed (Rv1196 or Rv1196_371-385_) irradiated BLCLs. Flow cytometry was performed for proinflammatory cytokine responses (IFN-g and TNF) to determine whether the Rv1196_371-385_ was presented to CD4 or CD8 T cells. A gating strategy was performed using live/dead exclusion, and this was followed by gating on singlets, lymphocytes, CD3^+^ events, and CD4^+^ events. (C) Flow plots showing the detection of DR, DP, and DQ alleles (colored according to table in C), following RM3 transfection. Limited expression can be observed in RM3 cells transfected with DPA/DPB (teal) and DRA/DRBw*2101 (orange) alleles, compared to untransfected RM3 cells. (D) The testing of three additional antibodies (antibody, clone, and fluorophore, listed above each graph), showing the detection of DPA/DPB allele expression in transfected RM3 cells. Download FIG S1, TIF file, 4.7 MB. Copyright © 2023 Grant et al. 2023 Grant et al. https://creativecommons.org/licenses/by/4.0/ This content is distributed under the terms of the Creative Commons Attribution 4.0 International license.

These data focused our efforts on producing M1-specific, MHC II allele-expressing RM3 cells, which is a cell line lacking MHC II expression (see Materials and Methods). We generated four sets of RM3-transfected cells, representing the dominant M1/M1 DR, DP, and DQ alleles (Fig. S1A). There was variable frequency in allele expression, despite within-protocol consistency, with the DP and DR alleles exhibiting lower frequencies of expression (Fig. S1C). After testing an alternative set of antibodies (different clones) for the detection of MHC II proteins, we observed a higher frequency of allele expression, indicating that our transfection experiments were successful and that available anti-DR/DP/DQ antibodies do not detect all alleles equally (Fig. S1D).

RM3-allele-expressing cells (RM3-DPA/DPB, RM3-DQA/DQB, etc.) were cocultured with expanded peptide-stimulated T cells to identify the specific M1 MHC II allele presenting Rv1196_371-385_ or Rv0125_81-92_. The frequencies of CD4 T cells producing IFN-γ and TNF were evaluated using flow cytometry staining under each of the following conditions: (i) PBMCs alone; (ii) RM3 cells with empty vector; (iii) RM3-DRA/DRB*w501; (iv) RM3-DRA/DRB*w2101; (v) RM3-DQA/DQB; and (vi) RM3-DPA/DPB in the presence of Rv1196_371-385_ or Rv0125_81-92_. This experiment was performed using frozen or fresh PBMCs from two Mtb-infected M1 MCMs for each specific peptide, and it revealed that both Rv1196_371-385_ and Rv0125_81-92_ are presented by the major DPA/DPB allele (Fig. 1H–K). This information was provided to the NIH tetramer core, where tetramers and monomers were prepared for DPA/DPB Rv1196_371-385_, DPA/DPB Rv0125_81-92_, and two previously published CFP-10 tetramers (i.e., DRA/DRB w501 CFP-10_36-48_ and DRA/DRBw501 CFP-10_71-85_) (3) for the investigation of samples from Mtb-infected MCMs.

### Mtb-specific CD4 T cells can be observed in the airways and blood of infected macaques.

To gain insight into the presence and function of Mtb-specific cells in M1 homozygous MCMs, 5 animals were infected with a low dose of Mtb and monitored throughout infection, using PET CT imaging, as previously described (43). Previous data showed that MCMs are more susceptible to Mtb infection than are Chinese cynomologus macaques; therefore, the study endpoint was planned for 10 weeks postinfection (36). However, the Mtb disease progressed faster in 3 of the animals, resulting in a clinical endpoint and necropsy at 6 to 9 weeks postinfection (Fig. S2A).

10.1128/mbio.00477-23.2 FIG S2 The animals used for tetramer^+^ CD4 T cell identification studies, the gating strategy for tissue samples from necropsy, and the flow reagents. (A) Table of MCMs used for the identification of tetramer^+^ cells in the periphery, BAL, and necropsy samples. (B) Flow cytometry gating tree for necropsy samples. Events were gated on live cells using Zombie NIR. Single cell events were selected from live events (FSC-A versus FSC-H). Lymphocytes were identified from single cells, based on FSC-A and SSC-A. Lymphocytes were gated based on CD3 and CD20 expression, with T cells (CD3^+^) being subsequently gated based on CD4^+^ and CD8a^+^ expression. All CD4^+^ cells were gated for tetramer^+^ events (the CD4 on the *x* axis and the tetramer on the *y* axis). All tetramer^+^ and tetramer^−^ cells were gated for transcription factors, granzyme B, or activation marker expression. Example: animal number 23620, RLL granuloma cluster 12. (C) Flow cytometry plots showing gating for Rv1196_371-385_ tetramer^+^ cells in tetramer, control tetramer, or no tetramer stained PBMCs at necropsy from animal number 23420. A gating strategy was performed using live/dead exclusion, and this was followed by gating on singlets, lymphocytes, CD3^+^ events, and CD4^+^ events, as described in panel B. (D) Table containing the antibodies used for staining the necropsy samples for the flow cytometry analysis. Download FIG S2, TIF file, 4.7 MB. Copyright © 2023 Grant et al. 2023 Grant et al. https://creativecommons.org/licenses/by/4.0/ This content is distributed under the terms of the Creative Commons Attribution 4.0 International license.

**FIG 2:**
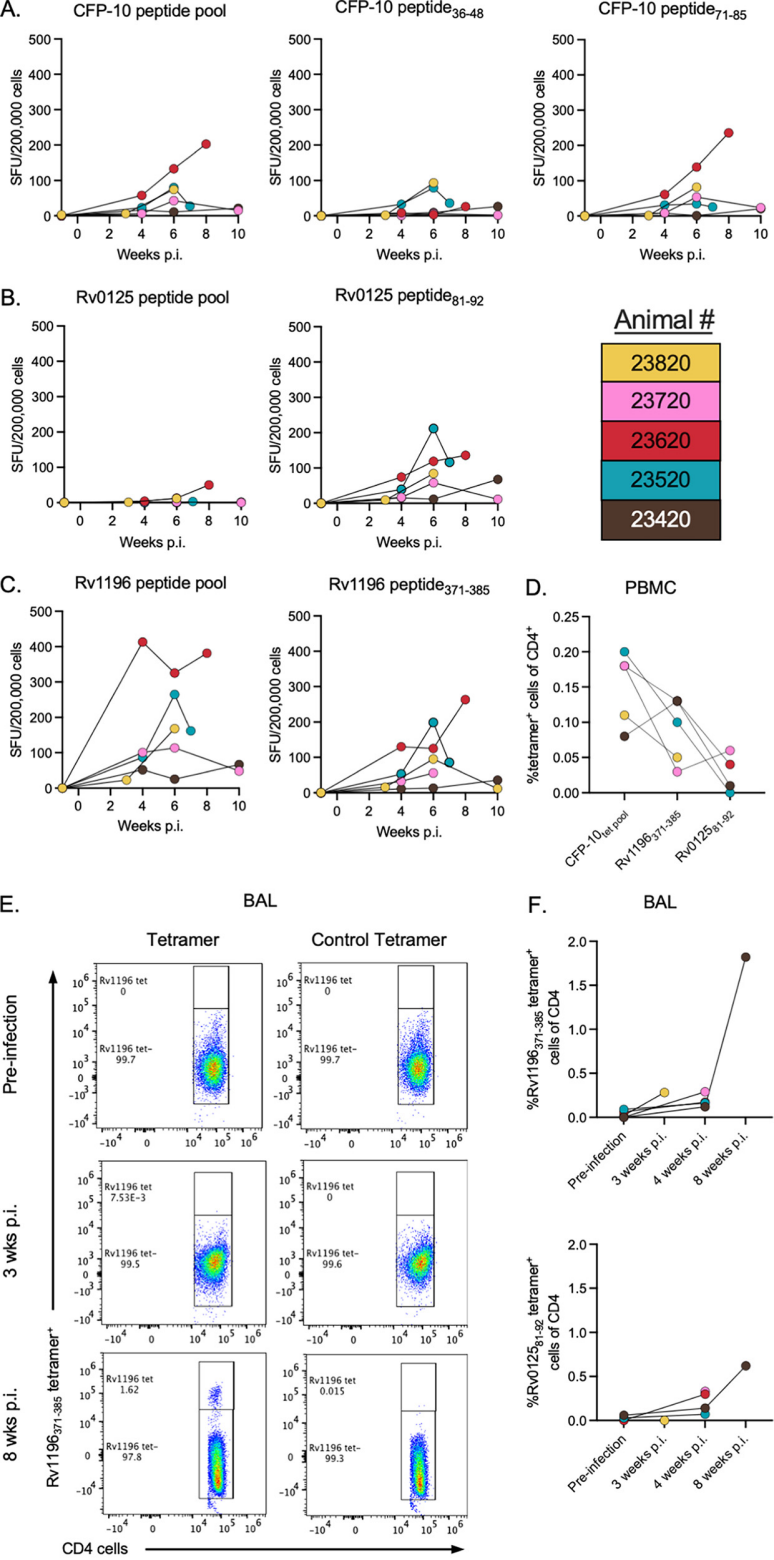
Identifying tetramer^+^ cells in the blood and BAL. IFN-γ ELISPOT response to (A) CFP-10 peptide pool, CFP-10_36-48_, and CFP-10_71-85_, (B) the Rv0125 peptide pool and Rv0125_81-92_, and (C) the Rv1196 peptide pool and Rv1196_371-385_ in PBMCs throughout an Mtb infection in 5 MCMs (weeks postinfection; color legend shown). (D) Frequency of tetramer^+^ cells of CD4^+^ cells in PBMCs at necropsy. (E) Flow cytometry plots showing control tetramer or Rv1196_371-385_ tetramer staining, gated on CD4 T cells, in preinfection and in 3 and 8 weeks postinfection BAL from animal number 23420. (F) Frequency of Rv1196_371-385_ tetramer^+^ CD4^+^ T cells (top) and Rv0125_81-92_ (bottom) in the BAL, both before and during infection.

Mtb-specific CD4 T cells in the blood were tracked throughout the infections via the monitoring of the IFN-γ responses that followed PBMC stimulation with dominant peptides (Fig. 2A–C). All animals responded to CFP10, Rv0125, and Rv1196 dominant peptides, although the responses were variable across animals and time points. At necropsy, PBMCs were stained with an optimized flow cytometry panel, including 4 tetramers that were specific for the 3 Mtb antigenic targets (CFP-10, Rv1196, and Rv0125) (Fig. 2C; Fig. S2D,). Although tetramer^+^ cells represent a low frequency of CD4 T cells in the blood of infected macaques (median values of 0.1 to 0.18%), a distinct population of Mtb-specific cells was observed in the blood at necropsy (Fig. S2D).

To monitor the appearance of tetramer^+^ cells in the airways, flow cytometry was performed on cells obtained via BAL, beginning at 3 to 4 weeks and, in one animal, at 8 weeks postinfection. There was an increase in the frequency of tetramer^+^ cells as the infections progressed (Fig. 2E and F). The adaptive immune response to Mtb is slow to evolve in macaques (and humans), which could account for the low frequency of Mtb-specific T cells observed in the airways at 3 and 4 weeks postinfection (44). Given the low numbers of tetramer^+^ cells in the BAL samples, further analyses on cell function were not performed.

### Identifying Mtb-specific CD4 T cells in thoracic lymph nodes, lungs, and granulomas.

**FIG 3:**
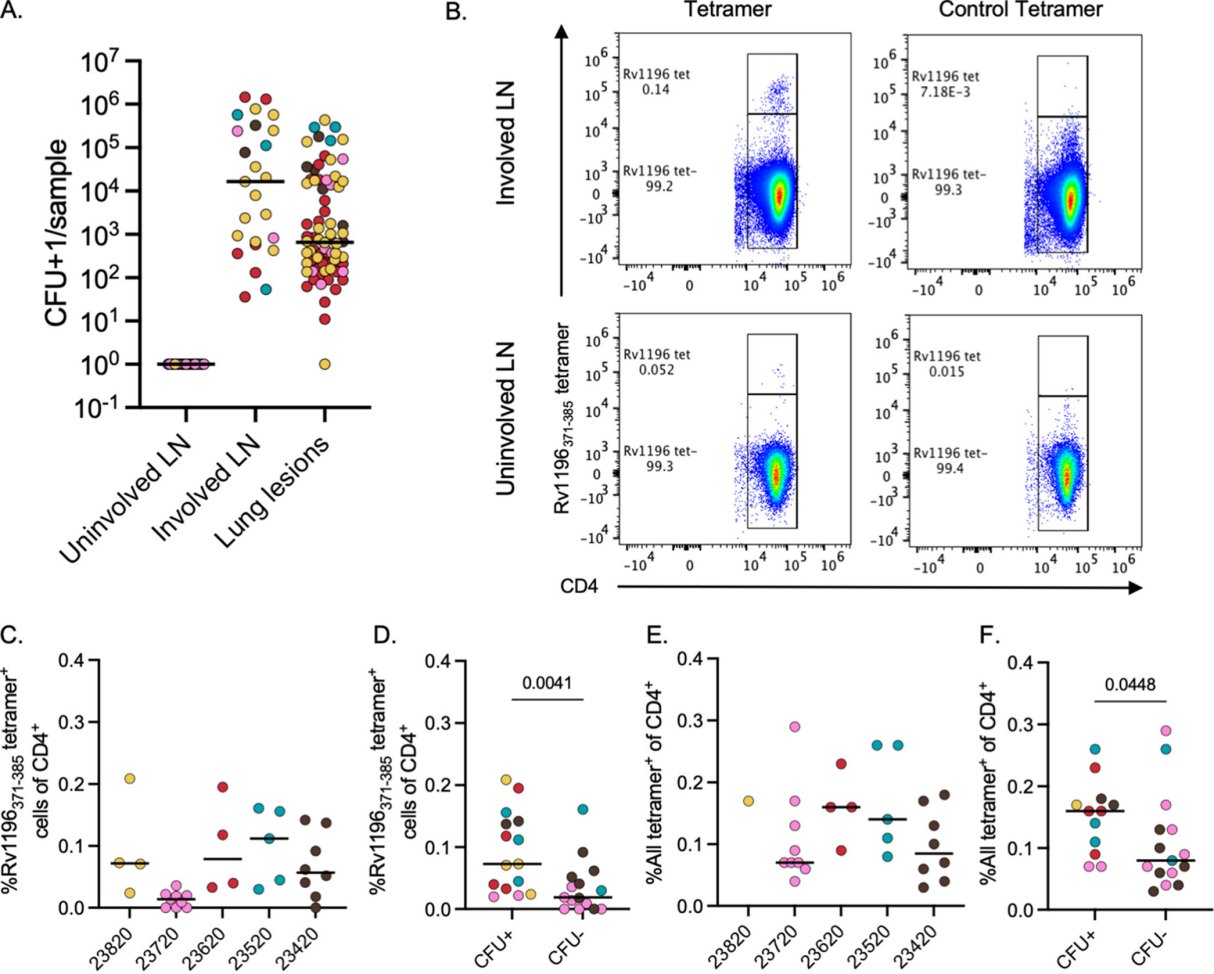
Detection of tetramer^+^ cells in thoracic LNs. (A) Mtb bacterial burden in uninvolved thoracic LNs (CFU-negative or no gross granuloma detected, *n* = 21), involved thoracic LNs (CFU-positive or the gross detection of granuloma, *n* = 25), and lung lesions (individual granulomas, clusters, and consolidations, *n* = 78), colored by animal (color legend as in Fig. 2). (B) Example flow cytometry plots, identifying control tetramer^+^ or Rv1196 tetramer^+^ cells in involved thoracic LN and uninvolved thoracic LN (monkey 23420). (C) Frequency of Rv1196 tetramer^+^ cells of CD4 T cells in thoracic LNs by animal (monkey 23820, *n* = 4; 23720, *n* = 9; 23620, *n* = 4; 23520, *n* = 5; 23420, *n* = 8). (D) Comparison of Rv1196 tetramer^+^ cells of CD4^+^ T cells in CFU^+^ (*n* = 15) and CFU^−^ (*n* = 15) thoracic LNs. (E) Results of Boolean gating for all tetramer^+^ cells of CD4^+^ T cells in thoracic LNs, by animal. Only samples for which all 4 tetramers were used are shown (monkey 23820, *n* = 1; 23720, *n* = 9; 23620, *n* = 4; 23520, *n* = 5; 23420, *n* = 8). (F) Comparison of all tetramer^+^ cells of CD4^+^ T cells in CFU^+^ (*n* = 12) and CFU^−^ (*n* = 15) thoracic LNs. Mann-Whitney tests were performed to compare the medians of CFU^+^ versus CFU^−^ thoracic LNs (panels D and F). The points represent individual samples, colored according to the animal. The bars represent medians.

One of the gaps in knowledge is the frequency and functionality of Mtb-specific T cells within the lungs, lymph nodes (LNs), and granulomas in NHPs or humans. The primary site of Mtb infection is the lung granuloma; however, a significant amount of disease, including the formation of granulomas, occurs within thoracic LNs (45). In this study, the median bacterial burden for involved LNs (i.e., those that were CFU positive) was higher than the median CFU for lung lesions (granulomas, clusters, and consolidations) (median for involved LN, 1.65 × 10^4^ CFU; median for lung lesions: 6.5 × 10^2^) (Fig. 3A). Given the high bacterial burden and the presence of granulomas in involved LNs, we hypothesized that Mtb tetramer^+^ cells could also be found within these sites. Using thoracic LN samples that were isolated at necropsy, we were able to identify Rv1196 tetramer^+^ cells within both involved LNs (CFU positive [CFU^+^] or the gross detection of granuloma formation) and uninvolved LNs (CFU negative [CFU^−^] and lacking the gross detection of granulomas) (Fig. 3B). There was a range in frequency of Rv1196_371-385_ tetramer^+^ cells within thoracic lymph nodes between 0.0% and 0.21%, with CFU^+^ LNs having a significantly higher frequency of Rv1196 tetramer^+^ cells, compared to CFU^−^ LNs (CFU^+^ median, 0.073%; CFU^−^ median, 0.019%; *P* = 0.0041) (Fig. 3C and D). To identify the frequency of all tetramer^+^ cells in thoracic LN samples (i.e., using all 4 tetramers), we applied a Boolean OR gating strategy which combines the events from the individual tetramer^+^ gates. This was only performed on a subset of samples for which we were able to use all 4 tetramers. The frequency of all tetramer^+^ cells reflected the frequency ranges of the Rv1196_371-385_ tetramer^+^ cells, providing a wider range for animal 23720, revealing that this animal either did not have many Rv1196_371-385_ tetramer^+^ cells or had poor Rv1196_371-385_ tetramer staining (Fig. 3E). As with Rv1196_371-385_ tetramer^+^ cells, there were significantly higher frequencies of all tetramer^+^ cells in thoracic CFU^+^ LNs, compared to thoracic CFU^−^ LNs (Fig. 3F).

**FIG 4:**
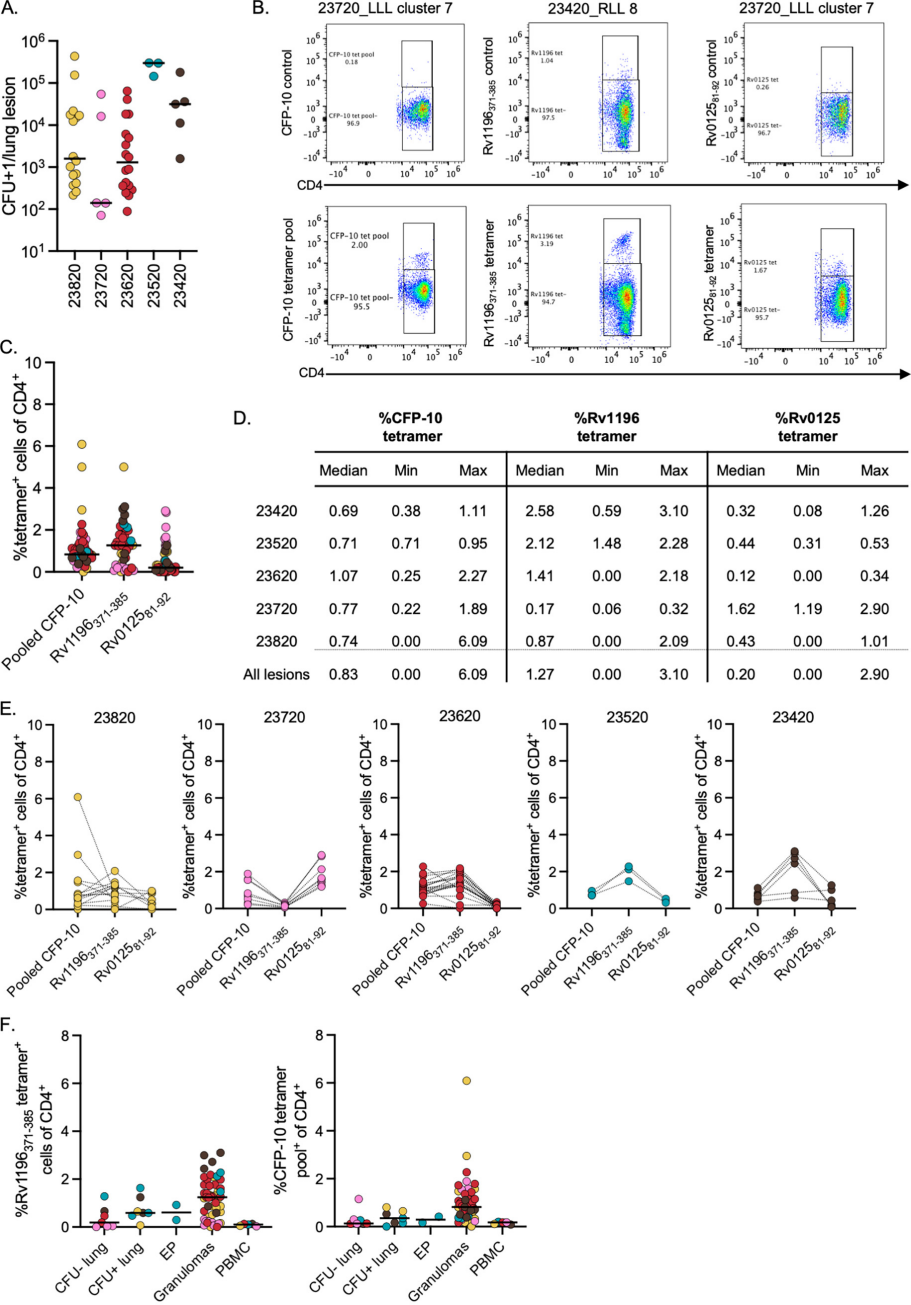
Identification of tetramer^+^ cells in lung lesions. (A) The range in bacterial burden per lung lesion in each macaque. The bars represent median values (monkey 23820, *n* = 16; 23720, *n* = 5; 23620, *n* = 18; 23520, *n* = 3; 23420, *n* = 5). (B) Representative flow cytometry plots showing the frequency of pooled CFP-10 tetramer^+^ cells, Rv1196_371-385_ tetramer^+^ cells, and Rv0125_81-92_ tetramer^+^ cells in CD4^+^ T cells in lung lesions. (C) Frequency of each tetramer^+^ population of CD4^+^ T cells in lung lesions (individual points, *n* = 46), colored by animal. The bars represent median values. (D) Median, minimum, and maximum frequencies for CFP-10, Rv1196, and Rv0125 tetramer^+^ cells among all CD4^+^ T cells for each macaque. (E) Frequencies of individual tetramer^+^ cells of CD4^+^ T cells within each animal. Lines connect the same lung lesion sample (animal number stated above each graph; monkey 23820, *n* = 15 for Rv1196 and CFP10, and *n* = 8 for Rv0125; 23720, *n* = 8 for all tetramers; 23620, *n* = 19 for all tetramers; 23520, *n* = 3 for all tetramers; 23420, *n* = 6 for all tetramers). (F) Frequency of Rv1196_371-385_ tetramer^+^ cells and CFP-10 tetramer^+^ cells in CFU^−^ lung samples (*n* = 7), CFU^+^ lung samples (*n* = 7), extrapulmonary (EP) granulomas (liver, *n* = 1; spleen, *n* = 1), lung granulomas (*n* = 51), and necropsy PBMCs (*n* = 5).

A total of 51 lesions (granulomas or granuloma clusters, individual or pooled) were analyzed for this study, with a the bacterial burden ranging between 7 × 10^1^ and 4.3 × 10^5^ CFU (Fig. 4A). The frequency of tetramer^+^ cells was variable in granulomas as well as within and across animals, with a median frequency of 0.83% of CD4 T cells staining positive for CFP-10 tetramers and a median frequency of 1.27% of CD4 T cells staining positive for the Rv1196_371-385_ tetramer; in contrast, the median frequency of Rv0125_81-92_ tetramer^+^ CD4 T cells was relatively low at 0.20% (Fig. 4B–E) (example flow cytometry plots in Fig. S2–S5). Comparing the frequency of individual tetramer staining within the same sample, there did not appear to be granulomas that had consistently high frequencies of every tetramer, but, instead, specific tetramer frequency varied within granulomas and across animals (Fig. 4E), suggesting that T cells of different antigen specificities were present across granulomas, even in the same animal, and this was most easily seen in animal 23820 (gold plot, Fig. 4E). These results also suggest that individual animals had variable T cell responses to these antigens.

As anticipated, when comparing the frequency of the tetramer^+^ cells in the blood to the site of infection (e.g., lung granulomas), there was an approximately 12-fold increase in Rv1196 tetramer^+^ cells in granulomas (median PBMC, 0.1%; median lung granulomas, 1.24% Rv1196) (Fig. 4F). There was a sixfold increase in the frequency of tetramer^+^ cells in lung granulomas, compared to uninvolved lungs (CFU = 0) (uninvolved lungs, 0.19%; median in lung granulomas, 1.24%) or even CFU^+^ lung samples (without obvious granulomas) (Fig. 4F). Although fewer samples were available, extrapulmonary granulomas (liver or spleen) were also observed to have low frequencies of Rv1196 tetramer^+^ cells (Fig. 4F). Similar trends were observed when comparing frequencies of CFP-10 tetramer^+^ cells (Fig. 4F). The Rv0125 tetramer was more limited in quantity. For granulomas, the frequency of Rv0125 tetramer^+^ cells was often low. Thus, Rv0125 tetramer^+^ cells were not included in this analysis. These data support the claim that lung granulomas are enriched sites for tetramer^+^ cells.

### Transcription factor and activation marker expression in tetramer^+^ lung granuloma cells.

To assess the potential phenotypic and functional differences between tetramer^+^ and tetramer^−^ CD4 T cells, granuloma samples were stained with the lineage-specifying transcription factors T-bet, GATA3, Foxp3, RORγT, and RORα. Although transcription factor expression does not demonstrate function, it does indicate the potential of the transcription factor^+^ CD4 T cells to express certain effector molecules. However, it is more appropriate to assume that transcription factor expression indicates polarization toward a certain phenotype. Although MHC class I tetramers in other disease models have been used in conjunction with intracellular cytokine staining, few studies have used intracellular cytokine staining in coordination with MHC class II tetramers (46, 47). One of the primary reasons for this is the instability of the CD4 TCR on the cell surface, following TCR ligation (48). Most protocols strongly recommend the use of a protein kinase inhibitor (PKI), such as dasatinib, to stabilize the TCR in order to enhance the staining potential of these cells. However, PKIs limit T cell activation and cytokine secretion (49). Thus, we used antibodies that were specific for transcription factors as a surrogate for cellular functionality or polarization along with our Rv1196 and CFP10 tetramers in our flow cytometry panel for the analysis of the PBMCs, lungs, LNs and lung granulomas (Fig. S2B–D, S3–S5). For these analyses, Rv0125_81-92_ tetramer^+^ cells were excluded due to their relatively low frequencies in these samples.

10.1128/mbio.00477-23.3 FIG S3 Example flow cytometry gating of transcription factor and activation marker expression on CD4 T cells and on Rv1196 tetramer^+^ CD4 T cells from granulomas. From granuloma cluster RLL12, examples of flow cytometry gating on all CD4+ T cells (A) or Rv1196 tetramer+ CD4 T cells (B) for transcription factors or activation markers. Download FIG S3, TIF file, 4.6 MB. Copyright © 2023 Grant et al. 2023 Grant et al. https://creativecommons.org/licenses/by/4.0/ This content is distributed under the terms of the Creative Commons Attribution 4.0 International license.

**FIG 5:**
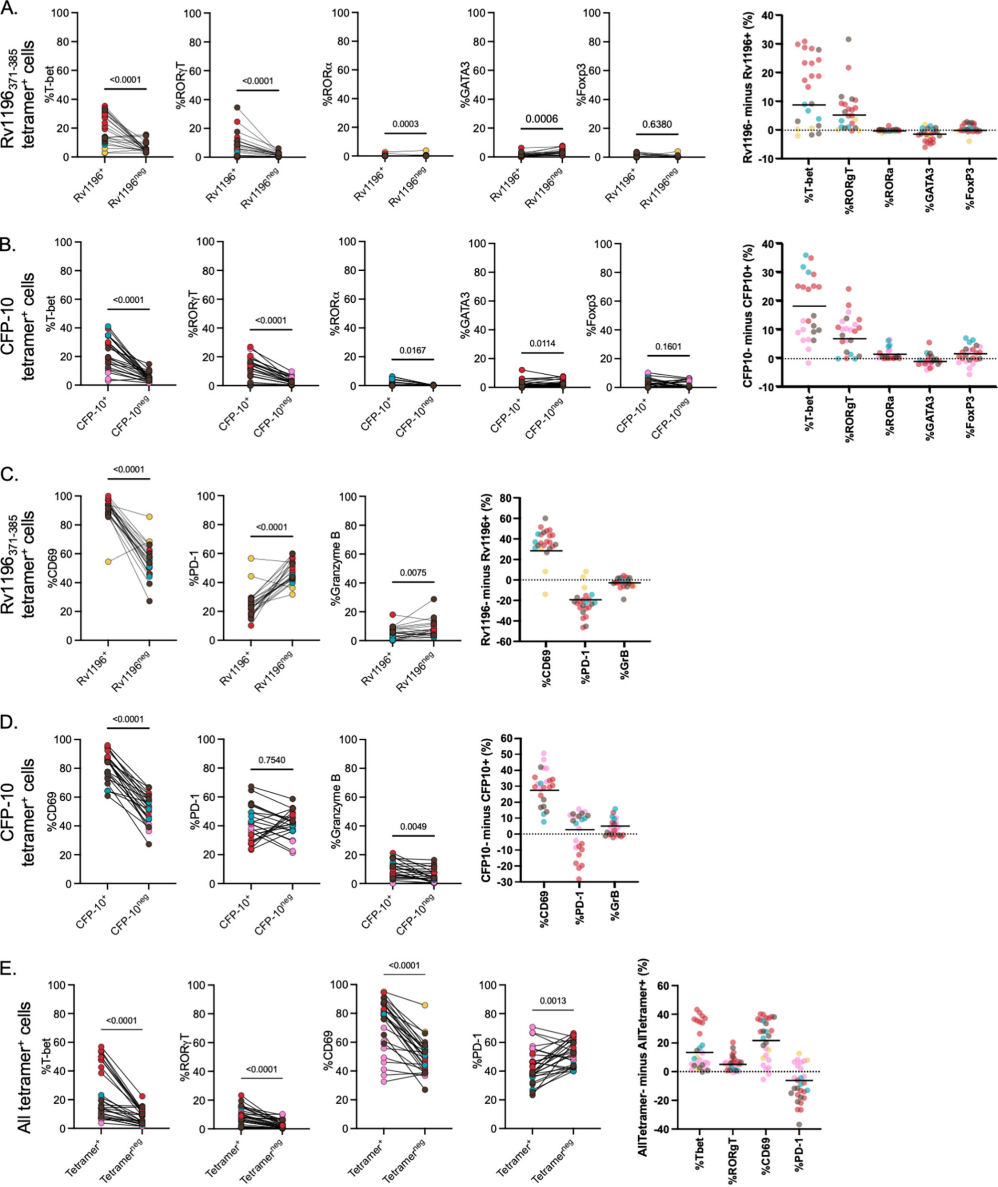
: Transcription factor and activation marker expression in tetramer^+^ granuloma CD4^+^ T cells. (A) Frequency of transcription factor expression in Rv1196_371-385_ tetramer^+^ CD4 T cells, compared to Rv1196_371-385_ tetramer^−^ CD4 T cells within the same granuloma sample (4 animals, *n* = 23 granulomas). Right panel: difference (tet^+^ − tet^−^) of each granuloma, colored by animal. The line represents the mean of the average number of granulomas from each animal. (B) Frequency of transcription factor expression in CFP-10 tetramer^+^ cells, compared to CFP-10 tetramer^−^ cells within the same granuloma sample (4 animals, *n* = 23 granulomas). Right panel: difference (tet^+^ − tet^−^) of each granuloma, colored by animal. The line represents the mean of the average number of granulomas from each animal. (C and D) Frequency of activation marker expression (CD69 and PD-1) and granzyme B in Rv1196_371-385_ tetramer^+^ CD4 T cells, compared to Rv1196_371-385_ tetramer^−^ CD4 T cells (panel C, 4 animals, *n* = 23 granulomas), and CFP-10 tetramer^+^ CD4 T cells, compared to CFP-10 pooled tetramer^−^ CD4 T cells (panel D, 4 animals, *n* = 23 granulomas) in granulomas. Right panel: difference (tet^+^ − tet^−^) of each granuloma, colored by animal. The line represents the mean of the average number of granulomas from each animal. (E) Frequency of T-bet, RORgT, CD69, and PD-1 expression in all tetramer^+^ cells, compared to tetramer^−^ cells, using Boolean OR gating for all 4 tetramers (5 animals, *n* = 28). Right panel: difference (tet^+^ − tet^−^) of each granuloma, colored by animal. The line represents the mean of the average number of granulomas from each animal. For all line plots, each dot represents an individual granuloma or pooled granulomas, colored by animal (color legend as in Fig. 2), with lines connecting the same sample for tetramer^+^ versus tetramer^−^ CD4 T cells. Statistical analyses were performed using the Wilcoxon matched pairs signed-rank test.

Significantly higher frequencies of T-bet and RORγT expression were observed for Rv1196_371-385_ and CFP-10 tetramer^+^ cells, compared to tetramer^−^ cells in the same sample (Fig. 5A and B). The frequencies for the remaining transcription factors were low in all CD4^+^ cells. Despite this, there was an overall trend of higher expression of GATA3 in tetramer^−^ cells, compared to tetramer^+^ cells. There was a significantly higher frequency of RORα expressing CFP-10 tetramer^+^ cells, compared to CFP-10 tetramer^−^ cells (Fig. 5B). There were no observable differences in the frequency of Foxp3 expression between tetramer^+^ and tetramer^−^ cells. Taken together with its low overall frequency, this result indicates that it is not highly expressed in CD4 T cells in MCM lung granulomas at the time points assessed (Fig. 5A and B).

We evaluated tetramer^+^ cells for activation marker expression (CD69 and PD-1) and for the production of the cytolytic molecule granzyme B. CFP-10 tetramer^+^ cells and Rv1196_371-385_ tetramer^+^ cells displayed significantly higher expression of CD69, compared to tetramer^−^ cells in the same sample, indicating that Mtb-specific CD4 T cells have an activated phenotype (Fig. 5C and D). When evaluated for PD-1, Rv1196_371-385_ tetramer^+^ cells had significantly lower frequencies of PD-1, compared to tetramer^−^ cells (Fig. 5C). This trend was not uniform when analyzing CFP-10 tetramer^+^ and tetramer^−^ cells but, instead, was animal dependent (Fig. 5D). The expression of granzyme B varied based on the sample and the specific tetramer, with significantly higher frequencies of granzyme B expression in CFP-10 tetramer^+^ cells, with the opposite being observed for Rv1196 tetramer^+^ cells (lower frequencies in tetramer^−^ cells), suggesting that each group of tetramer^+^ cells may differ in effector function (Fig. 5C and D). Despite variability in animal and tetramer, some tetramer^+^ cells express moderate amounts of granzyme B, with ranges between 0.0 and 18.0% for Rv1196_371-385_ tetramer^+^ cells and 0.0 to 21.2% for CFP-10 tetramer^+^ cells.

There were congruent results when using Boolean gating or individual gating, with higher frequencies of T-bet and RORγT in all tetramer^+^ cells than in tetramer^−^ cells in the same sample (Fig. 5E). Similarly, when CD69 and PD-1 expression were analyzed on all tetramer^+^ CD4 T cells, there were significantly higher frequencies of CD69 and lower frequencies of PD-1, compared to tetramer^−^ CD4 T cells within the same sample (Fig. 5E).

### Transcription factor expression in tetramer^+^ cells negatively correlates with the individual granuloma bacterial burden.

**FIG 6:**
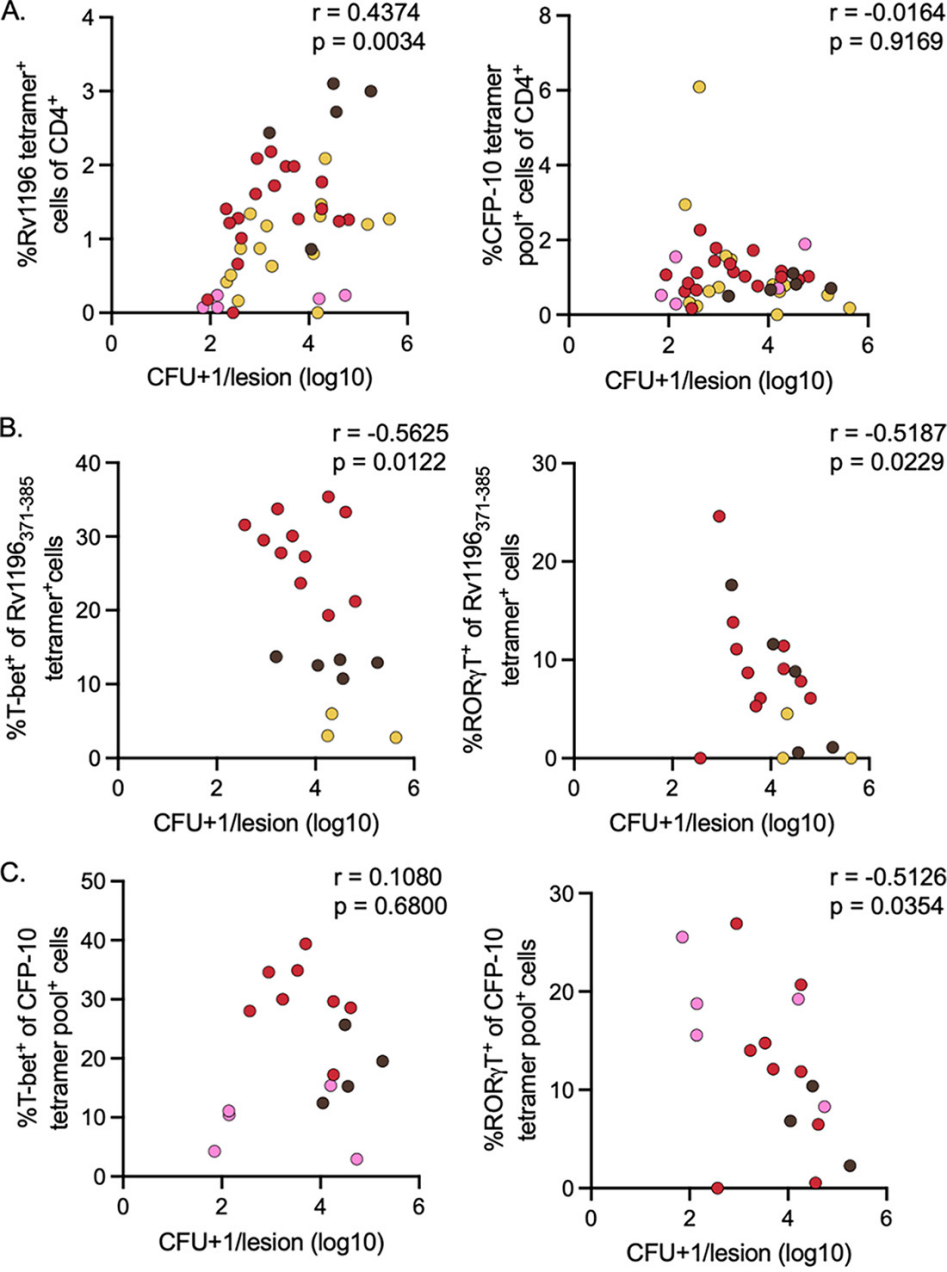
Transcription factor expression in tetramer^+^ cells negatively correlates with individual granuloma bacterial burden. (A) A modest but significant positive correlation was observed between the frequency of Rv1196 tetramer^+^ cells of CD4^+^ T cells and the log_10_ transformed CFU + 1/lesion value (left panel) (4 animals, *n* = 43 granulomas). No correlation was observed between the frequency of CFP-10 tetramer^+^ cells of CD4^+^ T cells and the log_10_ transformed CFU + 1/granuloma value (right panel, Spearman’s *r*, reported for nonparametrics) (4 animals, 43 granulomas). (B) Modest but significant negative correlations were observed between the frequency of T-bet (left) and RORgT (right) expression between Rv1196 tetramer^+^ CD4 T cells and the log_10_ transformed CFU + 1/granuloma value (3 animals, 19 granulomas). (C) No correlation was observed between the frequency of T-bet expression among CFP-10 pooled tetramer^+^ cells and the log_10_ transformed CFU + 1/granuloma value (left panel). A significant negative correlation was observed between the frequency of RORgT expression among CFP-10 tetramer^+^ cells and the log_10_ transformed CFU + 1/granuloma value (right panel) (*n* = 3 animals, 17 granulomas). Unless otherwise noted, Pearson’s *r* is reported.

To investigate the potential relationship between tetramer frequency and the bacterial burden within individual lesions, we compared the log_10_ CFU with the frequency of the Rv1196 tetramer^+^ or CFP-10 tetramer^+^ cells on an individual granuloma basis (i.e., only including individual isolated granulomas) (Fig. 6A). There was a modest but significant positive correlation (*r* = 0.4374, *P* = 0.0034) observed between Rv1196 tetramer^+^ CD4 T cells and CFU, whereas no correlation was observed between CFP-10 tetramer^+^ CD4 T cells and CFU (*r* = −0.0164, *P* = 0.9169) (Fig. 6A). In contrast, when transcription factor expression was included in the analysis, there was a modest but significant negative correlation observed between CFU and the frequency of T-bet (*r* = −0.5625, *P* = 0.0122) or RORγT (*r* = −0.5187, *P* = 0.0229) expression within Rv1196 tetramer^+^ cells (Fig. 6B). This modest negative correlation was also observed between CFU and the expression of RORγT within CFP-10 tetramer^+^ cells (*r* = −0.5126, *P* = 0.0354) but not the expression of T-bet (*r* = 0.1080, *P* = 0.6880) (Fig. 6C). For Fig. 6B and C, some granulomas (especially low CFU granulomas) could not be included in this analysis, as the parent gate (tetramer^+^ cells) did not have enough events for the appropriate analysis of transcription factors. Thus, the bacterial burden may drive increased numbers of Mtb-specific CD4 T cells in granulomas, but only polarized Mtb-specific CD4 T cells are associated with a reduced bacterial burden.

## DISCUSSION

Identifying Mtb-specific T cells in granulomas is critical to understanding the functional capabilities of these cells. Unlike viruses, Mtb expresses thousands of antigens, and studies in Mtb-infected humans demonstrate that T cells can recognize a wide range of these antigens with substantial variability among people (50). The full range of antigens recognized by T cells in granulomas is unknown. It is also likely that there are many T cells in granulomas that are not specific for Mtb but instead migrate to the granuloma due to inflammatory signals. Limiting the analysis to Mtb-specific T cells thereby provides a focused investigation of the function of these cells within granulomas. In this study, we developed tetramers that identify T cells that are specific for two Mtb antigens, namely, Rv1196 and Rv0125. We used these, in addition to two previously published CFP-10 tetramers (another Mtb antigen), to study the enrichment and phenotypes of these cells in lung granulomas and other tissue samples from Mtb-infected macaques. Data from our lab over the past two decades support that most Mtb-infected macaques have T cells that recognize these antigens. Although tetramers that are specific for Mtb antigens have been developed and used in mice for several years, mice do not form human-like granulomas, and the disease trajectory is dissimilar to that of humans. In contrast, NHP models have many similarities to human TB, including granuloma structure, other pathologies, and a wide range of Mtb infection outcomes. However, the rhesus and Chinese cynomolgus macaque MHC loci are extremely complex, even more so than the MHC in humans (35). Thus, we took advantage of the MCM model, which has relatively restricted and well-characterized MHC alleles, for our tetramer development and testing (29, 51, 52). Our data support the claim that granulomas are enriched for Mtb-specific CD4 T cells that have an activated Th1 or Th17 phenotype. Mtb-specific CD4 T cells expressing the key transcription factors T-bet or RORγT were associated with lower bacterial burdens in granulomas, thereby supporting the claim that these cells are performing functions that are critical to bacterial control.

MHC class II tetramers for the Mtb protein CFP-10 were described by collaborators (3) and were available through the NIH Tetramer Core. For the Mtb antigens Rv1196 and Rv0125, we identified the dominant epitope and MHC allele restriction. Rv1196 is a member of the PPE gene family, which is conserved across Mtb strains and M. bovis but is absent in other mycobacterial species (8). Though the function remains unknown, Rv1196 has been shown to induce IFN-γ responses and T cell proliferation in PBMCs from human PPD^+^ patients lacking evidence of TB disease, suggesting that it elicits a protective response (8). The second protein investigated in this study, namely, Rv0125, also elicits IFN-γ responses in PPD^+^ patients without evidence of TB disease (7). The predicted secreted serine protease Rv0125 is conserved across species within the Mtb complex and M. bovis but not in environmental mycobacteria (7). Given their conserved nature and ability to elicit IFN-γ responses within PPD^+^ protected individuals, Rv1196 and Rv0125 were included in the adjuvanted vaccine M72F, which has been shown to protect against the development of TB disease in subjects with asymptomatic Mtb infections and as a boost to the BCG vaccine in mice and rabbits (9, 53–57).

Here, we identified that the dominant epitope from both proteins was restricted by the MHC Class II *Mafa*-DPA1*07:02/*Mafa*-DPB1*19:03 alleles, which present antigens to CD4 T cells. The dominant epitope binding region, a.a. 371 to 385 in Rv1196, contained two amino acids, namely, valine and methionine (a.a. 383 and 384), which are necessary for eliciting an IFN-γ response. We did not observe this phenomenon with Rv0125 but determined that the optimal binding region is smaller than that of Rv1196. Using tetramers designed for these proteins and for the previously mapped CFP-10, we identified Mtb-specific CD4 T cells in various compartments, including the airways, uninvolved lung lobes, peripheral blood, thoracic LNs, and lung granulomas at necropsy. Comparing the frequencies of tetramer^+^ cells in uninvolved lungs or in the periphery with the frequencies observed in lung granulomas revealed that the granulomas are enriched for Mtb-specific (tetramer^+^) CD4 T cells.

The phenotype of Mtb-specific CD4 T cells in NHP TB lung granulomas had not been previously explored using tetramer staining. Here, we set out to identify a polarized or activated phenotype for tetramer^+^ cells using flow cytometry by staining for transcription factors, activation markers, and a cytolytic effector molecule. Two transcription factors that are associated with the T cell control of Mtb are T-bet and RORγT (44, 58, 59). T-bet is a transcription factor that induces several effector molecules, including the production of proinflammatory cytokines, cytotoxic effectors, and chemokines, as well as the regulation of T cell responses (60). In some studies, particularly in the context of vaccination, RORγT and the Th17 phenotype have been associated with protection against or the control of Mtb infections (58, 59, 61). Rv1196_371-385_ tetramer^+^ cells and CFP-10 tetramer^+^ CD4 T cells in granulomas had significantly higher frequencies of T-bet and RORγT expression, compared to tetramer^−^ cells. RORα, GATA3, and Foxp3 expression were relatively low in tetramer^+^ cells compared to T-bet or RORγT expression. These data reveal that Rv1196 and CFP-10 tetramer^+^ CD4 T cells exhibit a Th1 and/or Th17 phenotype. However, in both our study and others’ previous studies of NHP granulomas, whether by flow cytometry or single cell RNA sequencing, low frequencies of IL-17^+^ T cells were observed (20, 21, 62, 63). We previously surmised that the RORγT^+^ T cells might be ex-Th17 cells (62, 64).

There were significantly higher frequencies of CD69 expression on tetramer^+^ CD4 T cells, compared to tetramer^−^ CD4 T cells within the same granuloma. CD69 is a cell surface marker that is upregulated following T cell activation, although it also can play a role in cytokine release and cellular migration (65, 66). In contrast, PD-1 expression, which is a marker for both T cell activation and chronically stimulated cells, was significantly lower in Rv1196_371-385_ tetramer^+^ cells, compared to tetramer^−^ cells, although it varied within-animal for CFP-10 tetramer^+^ cells. This dynamic expression among tetramer^+^ cells of higher CD69 and lower PD-1 may indicate that these cells were not recently stimulated but instead express CD69 as a function of the immune environment and for its contribution to other effector functions. Although previous studies have shown low levels of exhaustion marker expression in T cells within NHP and human lung granulomas, this is the first direct evidence of low levels of PD-1 expression on tetramer-identified, Mtb-specific T cells in a macaque model (21, 23). Since granzyme B expression is more commonly associated with CD8 cytotoxic T cells, we expected low levels in the CD4 tetramer^+^ cells. However, since our gating included CD4^+^CD8^+^ T cells, we investigated the presence of granzyme B within tetramer^+^ cells and tetramer^−^ cells, and we found significantly higher levels of granzyme B expression within CFP-10 tetramer^+^ cells and lower frequencies in Rv1196 tetramer^+^ cells, suggesting that Mtb-specific cells for different antigens may have different functional capacities.

By comparing all of the tetramer^+^ cells to tetramer^−^ cells in individual granulomas, we observed higher frequencies of T-bet, RORγT, and CD69 as well as significantly lower levels of PD-1 in the tetramer^+^ cells. This reinforces that Mtb-specific T cells are activated and better poised for functionality in granulomas than are Mtb-nonspecific CD4 T cells. Although we only captured a small fraction of Mtb-specific CD4 T cells due to the large antigenic repertoire of mycobacteria, these data suggest that granulomas contain a population of Mtb-nonspecific CD4 T cells that migrate to the site of infection but are unlikely to participate in the control of an infection.

The variable frequency of tetramer^+^ T cells observed in lung granulomas both within and between animals /emphasizes the independent nature of granulomas. In addition, our data suggest that the recruitment of Mtb-specific cells is not solely based on optimal T cell recruiting granuloma phenotypes. For instance, we did not observe granulomas with higher frequencies of Rv1196_371-385_ tetramer^+^ cells also having higher frequencies of CFP-10 tetramer^+^ cells, as might be predicted, with the idea being that some granulomas may have a better capacity for Mtb-specific T cell recruitment through the production of chemokines or other cell signals. Rather, the data presented here suggest that T cells with different Mtb antigen specificities vary across granulomas, even within the same animal. One potential hypothesis for this observation is that the first Mtb-specific T cells that are recruited to a granuloma become the dominant specific cells through local proliferation. Also, the levels of tetramer^+^ cells within granulomas likely depend on the state of Mtb within those granulomas (i.e., quiescent, replicating, or high levels of dead bacteria). We observed a significant positive correlation between the frequency of Rv1196 tetramer^+^ cells and CFU in individual lesions. This suggests that as the bacterial burden increases, so does the recruitment or replication of specific T cells within individual lesions. However, we observed a significant negative correlation between the frequency of T-bet or RORγT within tetramer^+^ cells, suggesting that the functionality of Mtb-specific cells is critical for reducing the bacterial burden. We are not proposing that the T cells that recognize the specific antigens and express the transcription factors that were studied here are the only T cells that are associated with the control of infections. Instead, this study allowed us to focus on Mtb-specific T cells to only three antigens, as those are the antigens for which we were successful in making tetramers and for which we provide data supporting the claim that activated and polarized Mtb-specific CD4 T cells, in general, are associated with the control of infections. There are likely many Mtb antigens that are recognized by T cells in tissues, including granulomas, but we do not have tools with which to study all of the Mtb-specific T cells. In fact, our figures indicate that although there is an enrichment of transcription factors and activation markers in tetramer^+^ CD4 T cells in the tissues of infected macaques, it is clear that tetramer^−^ CD4 T cells can also produce these molecules, as would be expected, as at least some of the tetramer^−^ CD4 T cells must be specific for Mtb antigens.

Although the initial screening for epitopes was expected to identify both MHC class I and MHC class II epitopes, we only identified those recognized by CD4 T cells for both Rv1196 and Rv0125 proteins. This likely represents a limitation of our system, particularly in the use of IFN-γ as a functional readout, as CD8 T cells from NHP often express low levels of IFN-γ (20). In addition, CD4 T cells may outgrow the CD8 T cells in stimulated PBMC cultures. In the future, mapping peptides that elicit CD8 T cell responses could be achieved by modifying and optimizing the ELISPOT protocols for cytolytic molecules and peptide pools with 8 to 12 amino acids in length and by depleting CD4 T cells, prior to T cell culture. We identified limited frequencies of Rv0125 tetramer^+^ cells and, to some degree, CFP-10 tetramer^+^ cells in this study. Two potential hypotheses for this may be that Rv0125 is not highly produced by Mtb *in vivo* or that the binding affinity needed for the identification of tetramer^+^ cells is higher than that needed to elicit IFN-γ production. We also consistently observed low IFN-γ responses to the Rv0125 peptide pool in stimulated PBMCs, suggesting that this protein may contain other masking antigens (i.e., those that preferentially bind to MHC II molecules but do not elicit IFN-γ responses). One limitation to the use of tetramers in the context of a large bacterial pathogen (i.e., many proteins produced) is that it provides a narrow view, regarding the function of a small proportion of the potential specific cells. However, IFN-γ ELISPOTS performed using pools of 54 and 300 immunodominant peptides elicited similar levels of IFN-γ responses in NHPs, suggesting that there is a smaller set of Mtb proteins that are responsible for the majority of the IFN-γ Mtb response (2).

A final limitation of this study was the inability to stain for cytokines in conjunction with tetramers, given the need for the inclusion of dasatinib in the tetramer staining protocol to stabilize the MHC class II tetramer binding. Although we gained substantial information by including transcription factor staining, we were unable to assess the true effector functions of these Mtb-specific T cells. However, now that the tetramers are available, more advanced techniques, such as CITE-Seq may allow for the future transcriptional analyses of the functions of Mtb specific cells, thereby representing an additional advance in our understanding of T cell responses in granulomas.

In summary, our data demonstrate that granulomas are enriched sites for Mtb-specific CD4 T cells, compared to lung tissue, LNs, or blood. While we can identify tetramer^+^ cells in the airways as early as 3 weeks postinfection, the frequencies of cells are low and do not increase until 8 weeks postinfection. Within lung granulomas, our data revealed that the majority of Mtb-specific CD4 T cells are CD69^+^ Th1 cells or Th17 (or ex-Th17) cells. We demonstrated a modest but significant negative correlation between the expression of T-bet or RORγT within tetramer^+^ cells and the bacterial burden in granulomas, thereby highlighting the importance of polarized, Mtb-specific T cells in reducing the bacterial burden within lung granulomas. The use of tetramers provides insight into the phenotype of granuloma CD4 Mtb-specific cells, suggesting that enhancing or inducing Th1/Th17 functionality in Mtb-specific CD4 T cells would be advantageous in the context of vaccine development.

## MATERIALS AND METHODS

### Ethics statement.

All experiments, all protocols, and the care of the animals were approved by the University of Pittsburgh School of Medicine Institutional Animal Care and Use Committee (IACUC). The Division of Laboratory Animal Resources and IACUC adhere to national guidelines established by the Animal Welfare Act (7 U.S. Code Sections 2131 to 2159) and the Guide for the Care and use of Laboratory Animals (Eighth Edition), as mandated by the U.S. Public Health Service Policy. The animals used in this study were housed in rooms with autonomously controlled temperature, humidity, and lighting. Most animals were doubly housed (2 per large cage), although some were singly housed, based on temperament or uneven animal numbers in the study. The animals were provided with visual and tactile contact with neighboring conspecifics. The animals were provided water *ad libitum*, as well as large biscuits that were specifically formulated for NHPs and were supplemented with pieces of fresh fruits and vegetables at least 4 days per week. An enhanced enrichment plan was implemented with three components. First, species-specific behavior was encouraged, involving toys and other manipulata that were filled with food treats that were rotated on a regular basis. Puzzle feeders, foraging boards, and cardboard tubes encouraged foraging behaviors, and adjustable mirrors were accessible to the animals to stimulate interactions between the animals. Second, routine interaction between humans and macaques was encouraged. Interactions occurred daily and consisted of small food objects offered as enrichment. The interactions adhered to the established safety protocols. Animal caretakers were encouraged to interact with the animals while performing tasks in the housing area. Routine procedures were performed on a strict schedule to provide the animals with a routine daily schedule. Third, all macaques were provided with a variety of visual and auditory stimulation, including TV/video equipment playing cartoons for at least 3 h per day. These were rotated regularly so that the enrichment was not repetitively played for the same group of animals, as were the devices, including the food puzzles. All animals were checked twice daily and were closely monitored to assess appetite, attitude, activity level, hydration status, etc. Following the Mtb infections, the animals were monitored for evidence of disease (e.g., anorexia, weight loss, tachypnea, dyspnea, and coughing). Physical exams were performed on a regular basis. Ketamine or other approved drugs were used to sedate the animals prior to all veterinary procedures (e.g., blood draws, bronchoalveolar lavage). PET-CT imaging was conducted every other week for this study and has proven to be useful for monitoring the progression of disease. Veterinary technicians monitored the animals closely for signs of pain or distress. If any signs were noted, appropriate supportive care was provided (e.g., dietary supplementation), and clinical treatments were administered (analgesics). Any animal that was considered to have an advanced disease or intractable pain or distress from any cause was sedated with ketamine and was humanely euthanized using sodium pentobarbital.

### Animals, Mtb infection, and disease tracking by PET CT.

15 (10 for mapping and MHC allele testing and 5 for tetramer testing) Mauritian cynomolgus macaques (Macaca fascicularis) were obtained from Buckshire Corporation or Bioculture-Mauritius. The animals were placed in quarantine for either 30 or 60 days, depending on their length of time in the United States, and they were monitored for signs of poor health and prior infection. After quarantine, the animals were transferred to the University of Pittsburgh Regional Biocontainment Laboratory BSL3 facilities and infected with a low dose of (<20 CFU) Mtb Erdman via bronchoscopic instillation, as previously described (67, 68). The infection trajectory and disease were tracked using ^18^F-fluorodeoxyglucose (FDG) PET-CT for granuloma formation and lung inflammation every 4 weeks. PET CT scans were analyzed using OsiriX viewer with a 1 mm limit of detection, as previously detailed (43).

### Bronchoalveolar lavage (BAL).

Bronchoalveolar lavage was performed, as previously described (68). In brief, a 2.5 mm diameter bronchoscope was inserted into the trachea of a sedated animal and placed in the right middle of the lower lobe, where a saline solution (40 mL) was introduced and suctioned into a sterile 50 mL conical tube. An aliquot was plated on 7H11 agar plates to determine the CFU, which were counted after 3 weeks of incubation at 37°C and 5% CO_2_. The remaining BAL fluid was centrifuged at 1,800 rpm for 8 min at 4°C. The cells were resuspended in 1 mL of sterile PBS, counted using a hemocytometer, and aliquoted for use in tetramer and flow cytometry staining.

### Necropsy procedures.

Necropsies were performed on animals at the predetermined study endpoint or at the humane endpoint determined by a clinical evaluation or a PET CT scan. In brief, animals were sedated with ketamine, maximally bled, and then euthanized with pentobarbital, and tissue samples were extracted. PET CT-guided necropsy procedures were followed, as previously described, in which each granuloma or disease pathology was identified on the scan and matched to a lung location for dissection (43). All lung tissues, thoracic lymph nodes, spleens, and livers were also obtained. Individual samples were placed in RPMI 1640 media and homogenized for single cell suspensions. Sections of lymph node and lung tissue samples were formalin fixed for histopathological analysis. Single cell suspensions were counted using a hemocytometer to determine the viable cell counts, plated on 7H11 agar plates, and incubated for 21 days at 37°C for CFU determination. To increase the likelihood of capturing tetramer^+^ populations, smaller granuloma samples from the same animal were combined to increase the number of cells stained for a more distinct gating. Of note, granulomas from an individual animal were not combined with those from a different animal. Using this method, we were able to identify tetramer^+^ populations with >50 events in most of our pooled or individual granuloma samples. This allowed for sufficient cell numbers to enable the analysis of the transcription factor and other markers on tetramer^+^ CD4 T cells.

### IFN-γ ELISPOTS.

Peripheral blood mononuclear cells (PBMCs) were isolated from blood draws, both pre- and post-Mtb infection. White membrane plates (Fisher Scientific, catalog number MSIPS4W10) were prepared using 30% ETOH, and this was followed by three washes with sterile 1× PBS. Primary antibody (anti-IFN-γ clone MT126L) was used as a capture antibody, and, following its addition, the plates were incubated overnight at 37°C. Following the addition of the capture antibody, ELISPOT plates were washed three times with sterile 1× PBS and blocked for 2 h at 37°C using RPMI 1640 (Sigma, catalog number R0883) supplemented with 1% l-glutamine (Thermo Fisher, catalog number 25030149), 1% HEPES (Thermo Fisher, catalog number SH3023701), and 10% human A/B serum (Gemini Bio-Products, catalog number 100-512). Peptides were added to prepared plates at a final concentration of 1 μg/mL to 2 μg/mL, and this was followed by 1 × 10^5^ to 2 × 10^5^ fresh or frozen (rested overnight) PBMCs. The plates were incubated for 48 h at 37°C and immediately washed 6 times with 1× PBS. Following the washing, a filtered secondary antibody (anti-IFN-γ, clone 7-B6) was added to the plates and incubated for 2 h at 37°C. The plates were washed 6 times with 1× PBS. This was followed by the addition of a Streptavidin-HRP antibody for 45 min at 37°C. Last, an AEC substrate (Vector Laboratories, catalog number SK-4200) was added, according to the manufacturer’s instructions, for 5 to 8 min to allow the plate to develop. This was followed by 3 washes each of diH_2_0 and 1× PBS. All samples were run in duplicate wells for each experiment and were represented as the average spot forming unit (SFU). The plates were dried in a cool, dark location, read using an Immunospot ELISPOT plate reader (Cellular Technology Limited, Cleveland OH, USA), and manually assessed for quality control.

### Peptide resuspension and mapping strategies.

The sequences for the Mtb proteins Rv1196 and Rv0125 were obtained from publicly available databases. Peptide libraries were generated using a commercially available peptide library design tool (PEPscreen, Millipore Sigma, Merck, Darmstadt, Germany) with peptide lengths of 20 amino acids (a.a.) and an overlap of 10 a.a. Individual peptides were ordered from Genscript and were resuspended in DMSO (with a calculated final concentration of no greater than 10%) and sterile 1× PBS, according to their molecular weights, to obtain a stock concentration of 10 mM. The stock concentrations were diluted using sterile 1× PBS to 1 mg/mL working concentrations. Peptides were used at 1 to 4 μg/mL, according to assay standards.

### Plasmid generation and purification.

Plasmids containing major M1 allele variants (both MHC I and MHC II) (Fig. S1A) were kindly gifted from collaborators at the University of Wisconsin (O’Connor lab). To obtain larger stocks, plasmids were transformed into E. coli using heat shock transformation, selected on antibiotic plates, and grown in liquid media. Plasmids were purified from the E. coli strains using a Qiagen Miniprep Kit with a vacuum manifold. The concentrations of plasmids were determined using a NanoDrop reader with OD 260/280 ratios of approximately 1.8 being considered to be pure.

### Transfection of mammalian cells for allele expression.

Several transfection methods were tested, and success rates varied, based on the plasmids containing allele variants. Mammalian cells lacking MHC I (K562 cells) or MHC II (RM3 cells) proteins (kindly sent by Shelby O’Connor) were used for allele restriction determination. Electroporation using a BTX ECM630 was determined to be the optimal system for the transfection of RM3 cells containing MHC II allele variants under the following conditions: mode, low voltage; capacitance, 250μF; resistance, none; charging voltage, 200 V; chamber, BTX disposable cuvette (2 mm gap); field strength, 750 V/cm; sample volume, approximately 300 μL (3, 69–71). Prior to electroporation, the RM3 cells were kept in a log growth phase by subculturing and observing flasks for cell health and debris. Plasmids were added to cuvettes (10 μg of total plasmid; [e.g., 5 μg of DRA and 5 μg of DRB]) along with RM3 cells that were resuspended in 150 mL of ECM buffer. Following electroporation, RM3 cells were grown under unsupplemented media for 24 h at 37°C and 5% CO_2_. After 24 h, the medium was changed, and the cells were grown in R10 medium (RPMI 1640 + 10% FBS, 1% antimycotic/antibiotic) for 48 h. At this time, hygromycin B (Sigma, catalog number H0654-500mg) was added to the media at a final concentration of 400 μg/mL for an additional 6 days, with subculturing every 48 to 72 h. The transfectants were stained for viability using a live/dead blue dye, and this was followed by anti-HLA DR DP DQ antibodies (Bio-Rad clone Bu26, catalog number MCA2497F; Beckman clone I3, catalog number CO6604366; BioLegend, clone Tü39, catalog number 361704) or anti-HLA DP (Leinco, clone B7/21, catalog number H129) for the detection of MHC II proteins.

### Generation of EBV transformed-B lymphoblastic cell lines (BLCLs).

Autologous BLCLs were generated using isolated PBMCs from preinfection blood draws for use as antigen-presenting cells and as culturing and testing T cell lines. Isolated PBMCs (1 × 10^5^ to 2 × 10^5^) were added to 96-well round-bottom plates with 50 μL of Herpesvirus papio supernatant from filtered supernatants of S594 cells, and they were grown in R20 (RPMI + 20% FBS). The medium was changed 3 to 4 days after the addition of the S594 supernatant as well as every 2 to 3 days, thereafter. When the cells started to clump and the medium was slightly yellow, the BLCLs were split into additional wells of the 96-well plate. Cells from the 96 wells were combined into wells of a 24-well plate in R20 and expanded into larger size wells (a 6-well plate) with T25 or T75 flasks being seeded for freezing or for use in experiments.

### Peptide-specific T cell generation and expansion.

A series of experiments were performed to generate T cells for long term culture and testing, according to protocols provided by the O’Connor lab as well as other published protocols (72). However, in our experiments, stimulation with peptide-pulsed irradiated BLCLs did not yield stable peptide-responsive T cell lines. We used the following approach for the data provided here. Isolated PBMCs were plated in 12-well or 6-well plates at a concentration of 1 × 10^6^ cells/mL in RPMI media supplemented with 15% FBS, IL-2 (Abcam, catalog number ab119439, 1 μL/10 mL) and IL-7 (Abcam, catalog number ab73201, 1 μL/20 mL). The peptide of interest was added to individual wells at a final concentration of 1 μg/mL peptide. The cells were incubated at 37°C and 5% CO_2_ for 48 h and were then washed with sterile 1× PBS and rested for an additional 4 days in R15+IL-2/IL-7 media.

### Peptide-specific T cell allele restriction testing.

PBMC T cell cultures expanded with the dominant epitope for RV1196 or Rv0125 were tested for specific allele presentation, using RM3 cells transfected with MHC II alleles at a 1:1 or 2:1 ratio (i.e., T cell:transfected RM3 cell) (Fig. S1). Transfected RM3 cells were prepared for T cell testing via the addition of 1 μg/mL peptide to individual wells of a 96-well plate and were incubated for 90 min at 37°C and 5% CO_2_. Following the incubation, the prepared T cells were added to each well of a 96-well plate and incubated for another 12 h in the presence of a Golgi plug inhibitor, namely, BFA (BD Biosciences, catalog number 555029). Stimulation plates were spun down and washed with 1× PBS, following 12 h of incubation. Flow cytometry staining was performed for viability (Live/Dead Fixable Blue Dead Cell Stain Kit, Thermo Fisher, catalog number L34962) and surface marker expression for CD3 (BD, clone SP34-2), CD4 (BD, clone L200), CD8 (BD, clone RPA-T8), HLA DR/DP/DQ (Bio-Rad clone Bu26; Beckman clone I3; BioLegend, clone Tü39), and the intracellular cytokines IFN-γ (BD, clone B27) and TNF (BD, clone Mab11), using standard flow cytometry and intracellular staining protocols. The samples were run on a BD LSRII or a Cytek Aurora (Cytek, Bethesda, MD, USA) and were analyzed using the FlowJo software package (BD, version 10).

### Tetramerization.

Monomers were prepared by the NIH tetramer core, as requested, for peptide and allele sequences. Once received, samples were aliquoted and stored at −80°C until the time of use. To tetramerize monomers, fluorescently labeled streptavidin (SAV) was added in 10-minute increments, and this occurred 10 times, based on the concentration of the SAV, as previously described in the NIH tetramer core published protocols (73). The serial addition of SAV allows for the binding of free monomer while leaving little excess SAV. After tetramerization, staining was performed on frozen PBMCs or on expanded T cells to ensure proper complex formation. The tetramers were stored at 4°C and were used prior to 2 months from synthesis to allow for minimal tetramer disaggregation. Tests were performed to determine the optimal amount of tetramer to use per sample by comparing control tetramer staining to Rv1196_371-385_ tetramer staining and showing that 4 μg/mL was adequate to visualize a distinct population of tetramer^+^ cells. For tetramer staining, samples were prepared, stained in a 500 nM RPMI+dasatinib (Fisher, catalog number NC0897653) solution, and washed with a 50 nM dasatinib solution in 1× PBS. The samples were stained with tetramer, control tetramer (CLIP), or no tetramer for 30 min at room temperature (RT), and this was followed by two washes with 50 nM 1× PBS. To maximize the use of validated fluorophores for tetramer conjugation, we conjugated monomers for CFP-10_36-48_ and CFP-10_72-85_ in the same fluor (BV421), as both detect T cell responses to the protein CFP-10, conjugated monomer for Rv1196_371-385_ with PE, and the monomer for Rv0125_81-92_ in APC. The concentration of tetramer needed for the clear identification of tetramer^+^ populations was determined using expanded T cell lines and was compared to control tetramer staining at the same concentration. Granuloma lung samples were stained with control tetramers (CLIP monomer provided by NIH and tetramerized by us), no tetramer, no transcription factor, or tetramers plus transcription factors, depending on cell numbers, with all of the controls being performed for each animal to ensure accurate gating for the analysis of tetramer^+^ and transcription factor^+^ cells.

### Flow cytometry and transcription factor staining.

Bronchoalveolar lavage was performed as described above, and cells were counted for staining at 3 and 4 weeks postinfection for all animals and at 8 weeks postinfection for 1 animal. Similarly, necropsy tissues were homogenized and counted, and single cell suspensions were prepared via washing with a 500 nM dasatinib RPMI solution. After the tetramer staining and washes were performed, the cells were stained with Zombie near infrared (NIR) (BioLegend, catalog number 423105) and diluted 1:1000, per the manufacturer’s recommendation, for 10 min at 4°C in the dark. The cells were washed with 50 nM dasatinib in 1× PBS twice, and they were spun down each time at 2,000 rpm for 3 min at 4°C. Following the washes, the cells were stained with a surface antibody cocktail diluted in 50 nM dasatinib FACS (1× PBS + 5% FBS) buffer for 20 min at 4°C in the dark. The cells were washed twice using a 50 nM dasatinib FACS solution, spun as described above, and fixed for a minimum of 10 min in 4% paraformaldehyde (PFA) for processing outside the BSL3. The samples were washed in 1× PBS and placed at 4°C overnight for continued staining on the following day. To begin the staining on the following day, the samples were spun at 2,000 rpm for 3 min at 4°C, and the supernatants were decanted. A True Nuclear Transcription Factor Buffer Kit (BioLegend, catalog number 424401) was used for the detection of transcription factors within cells, per the manufacturer’s recommendation. Following a 1 h permeabilization at RT in the dark and two subsequent washing steps, the samples were stained for 1 h at RT in the dark, using antibodies targeting transcription factors, granzyme B, and CD69. The samples were washed twice using a 50 nM dasatinib FACS solution and resuspended in 1× PBS. The granuloma lung samples were stained with control tetramers (CLIP monomer provided by NIH and tetramerized by us), no tetramer, no transcription factor, or tetramers plus transcription factors, depending on the cell numbers, with all of the controls being performed for each animal to ensure accurate gating for the analysis of tetramer^+^ and transcription factor^+^ cells. For the analysis, we employed a gating strategy, using an inclusive CD4 gate (gated on all CD4^+^ cells, including CD4^+^CD8^+^) to optimize our ability to identify the tetramer^+^ cells (Fig. S2–4). In addition, we demonstrate that staining with multiple tetramers on the same sample gave minimal costaining of individual cells with more than one tetramer (Fig. S5). A Cytek Aurora was used to acquire sample events, and the analysis was performed on unmixed data files (adjusted for reference controls), using FlowJo version 10.

10.1128/mbio.00477-23.4 FIG S4 Example flow cytometry gating of transcription factor and activation marker expression on CFP10 tetramer^+^ and on Rv0125 tetramer^+^ CD4 T cells from granulomas. From granuloma cluster RLL12, examples of flow cytometry gating on CFP10 tetramer^+^ CD4^+^ T cells (A) or Rv0125 tetramer^+^ CD4 T cells (B) for transcription factors or activation markers. Download FIG S4, TIF file, 4.6 MB. Copyright © 2023 Grant et al. 2023 Grant et al. https://creativecommons.org/licenses/by/4.0/ This content is distributed under the terms of the Creative Commons Attribution 4.0 International license.

10.1128/mbio.00477-23.5 FIG S5 Minimal costaining of tetramers on the same granuloma sample. The granuloma cluster RLL 7 was stained with all tetramers to determine whether there was multiple tetramer binding to the same cell. Top panel: flow plots showing the minimal costaining of cells with more than one tetramer, using each tetramer combination. Bottom panel: flow plots of each tetramer, showing positive tetramer staining on CD4 T cells. Download FIG S5, TIF file, 4.0 MB. Copyright © 2023 Grant et al. 2023 Grant et al. https://creativecommons.org/licenses/by/4.0/ This content is distributed under the terms of the Creative Commons Attribution 4.0 International license.

### Statistical analysis.

The statistical analysis was performed in GraphPad Prism (version 9). The data were tested for normality using the Shapiro-Wilk test. Groups were compared using a Wilcoxon ranked-sum analysis for paired samples or a Mann-Whitney test for unpaired samples. For correlations, the CFU (+1) was log_10_-transformed and tested for normality. For normal data, the Pearson’s *r* correlation coefficient was reported. Otherwise, the Spearman’s *r* correlation coefficient was reported. A *P* value of <0.05 were considered to be indicative of a statistically significant result.
