# Supplementary figures and images for: Mycobacterium tuberculosis-Specific CD4 T Cells Expressing Transcription Factors T-Bet or RORγT Associate with Bacterial Control in Granulomas
Source: mBio. 2023 Apr 11;14(3):e00477-23. doi: 10.1128/mbio.00477-23 (PMC10294621; doi:10.1128/mbio.00477-23)

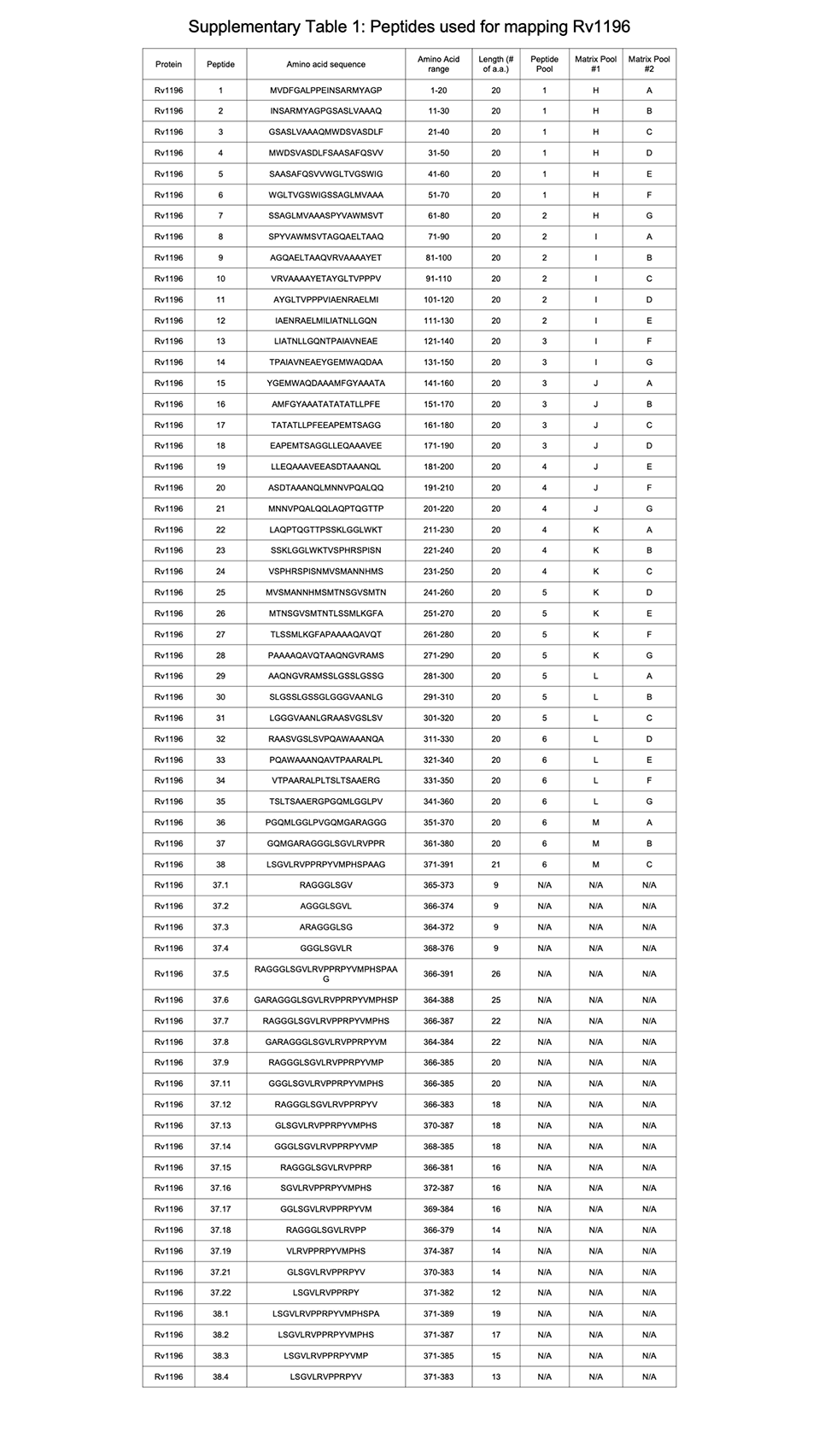

Supplement: TABLE S1 [file mbio.00477-23-s0006.tif]

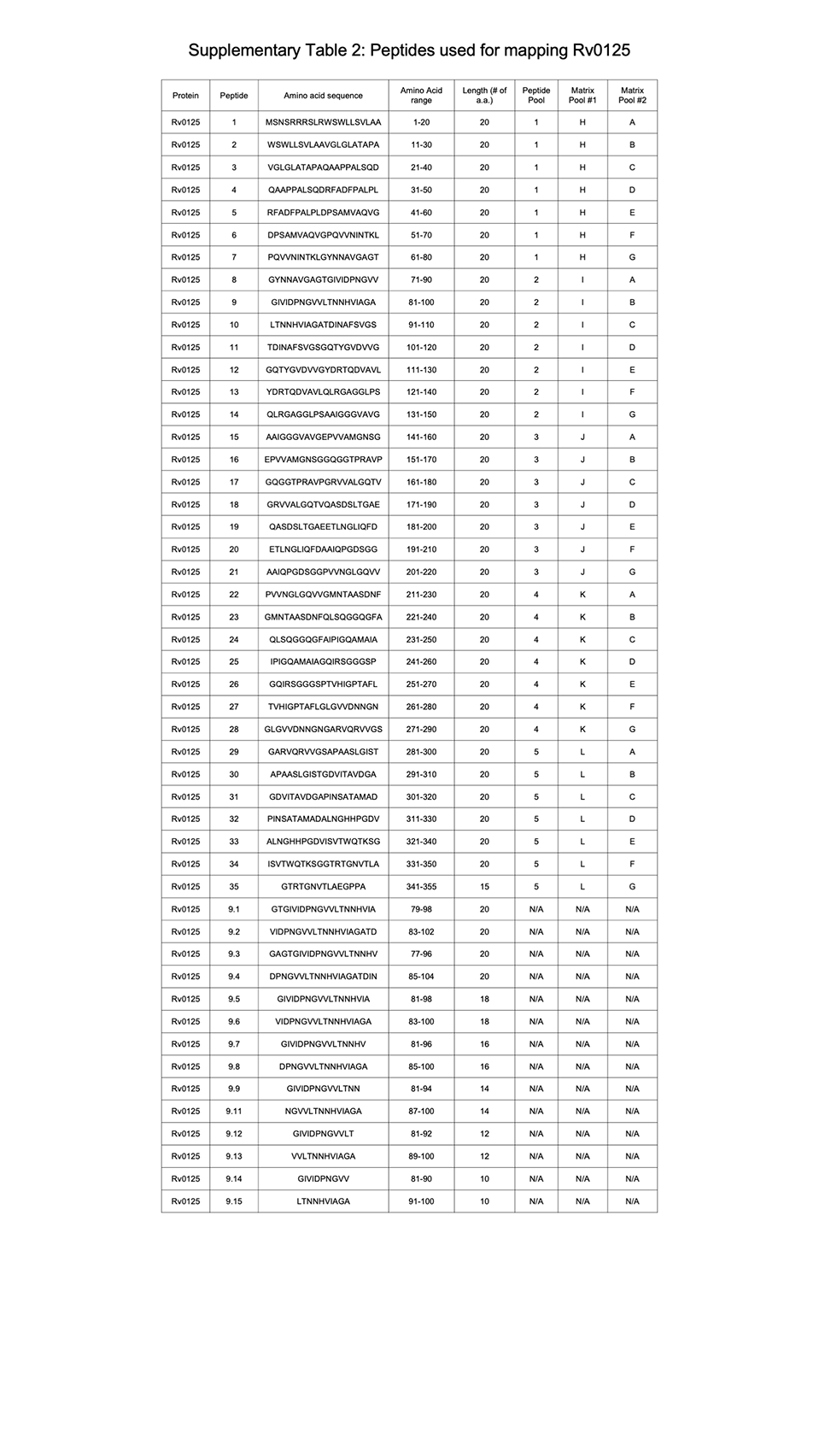

Supplement: TABLE S2 [file mbio.00477-23-s0007.tif]

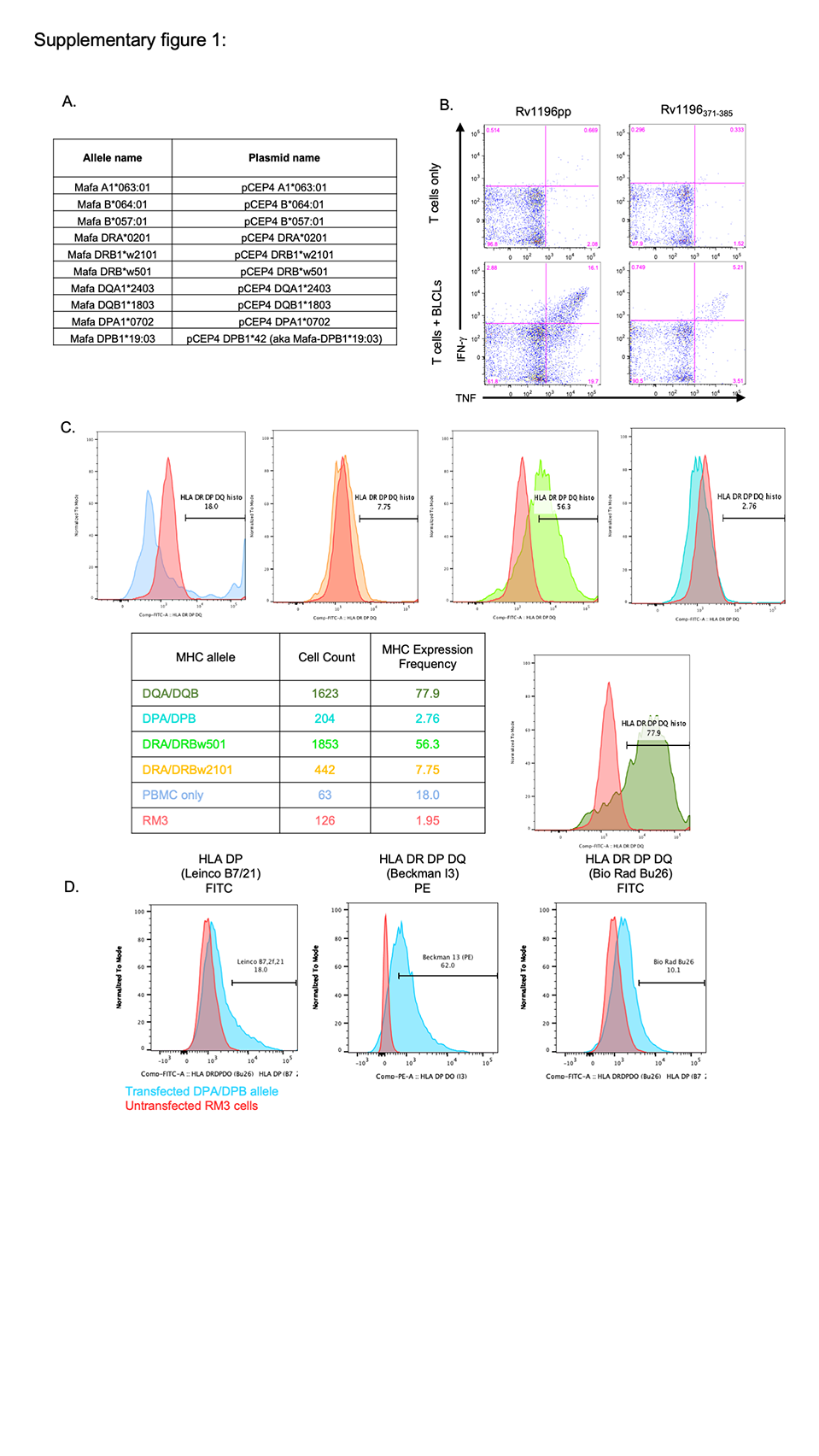

Supplement: FIG S1 [file mbio.00477-23-s0001.tif]

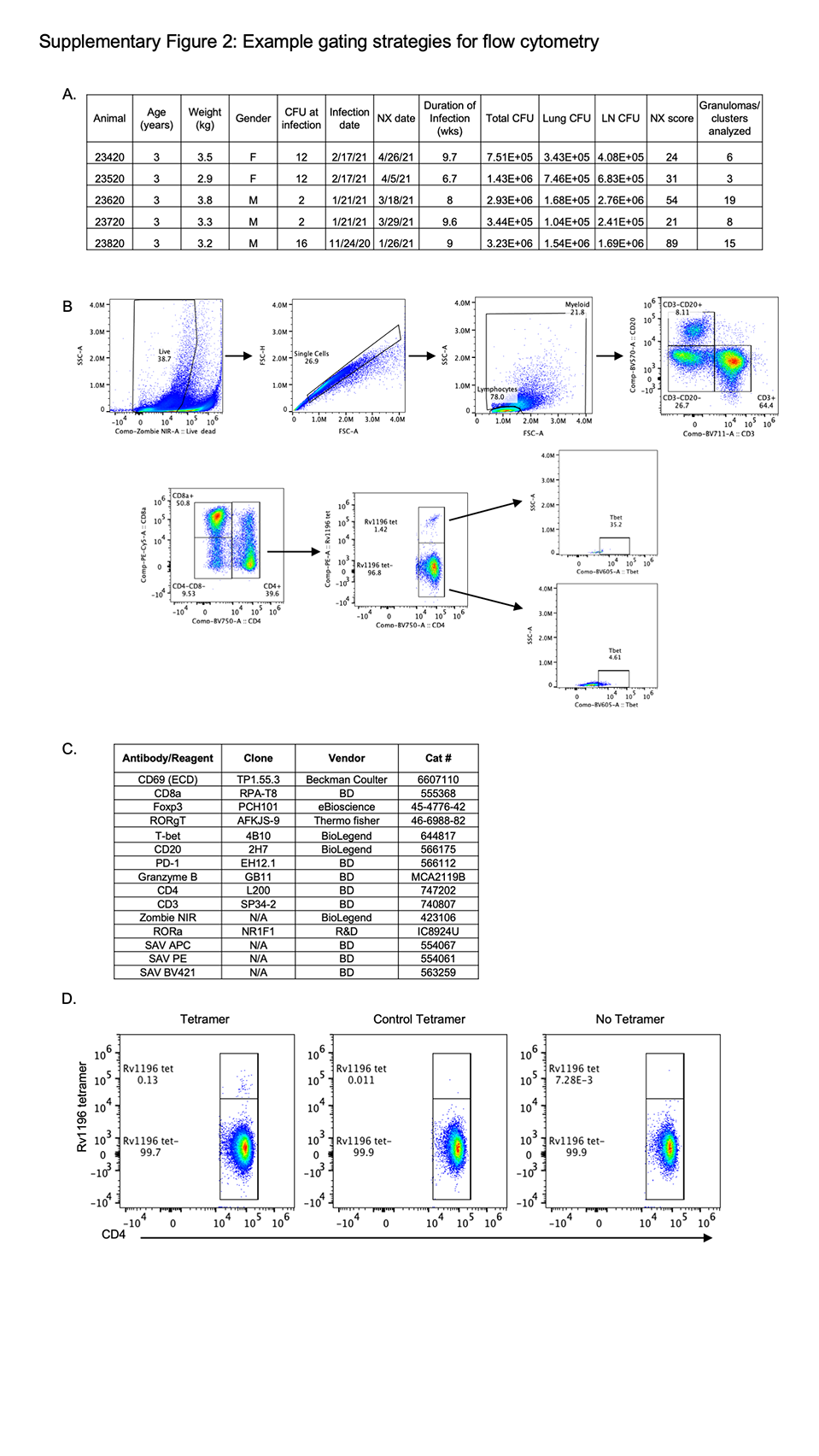

Supplement: FIG S2 [file mbio.00477-23-s0002.tif]

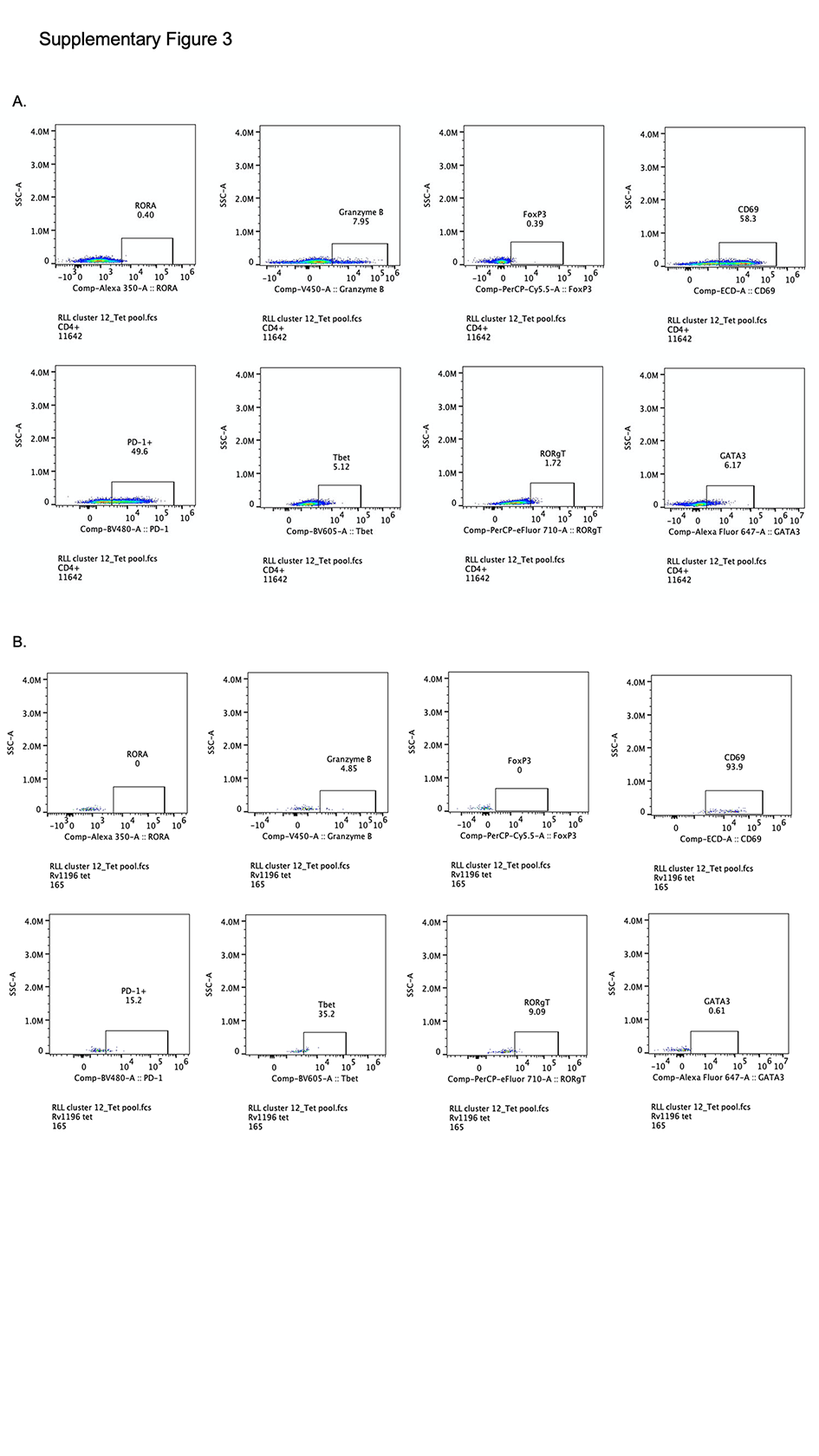

Supplement: FIG S3 [file mbio.00477-23-s0003.tif]

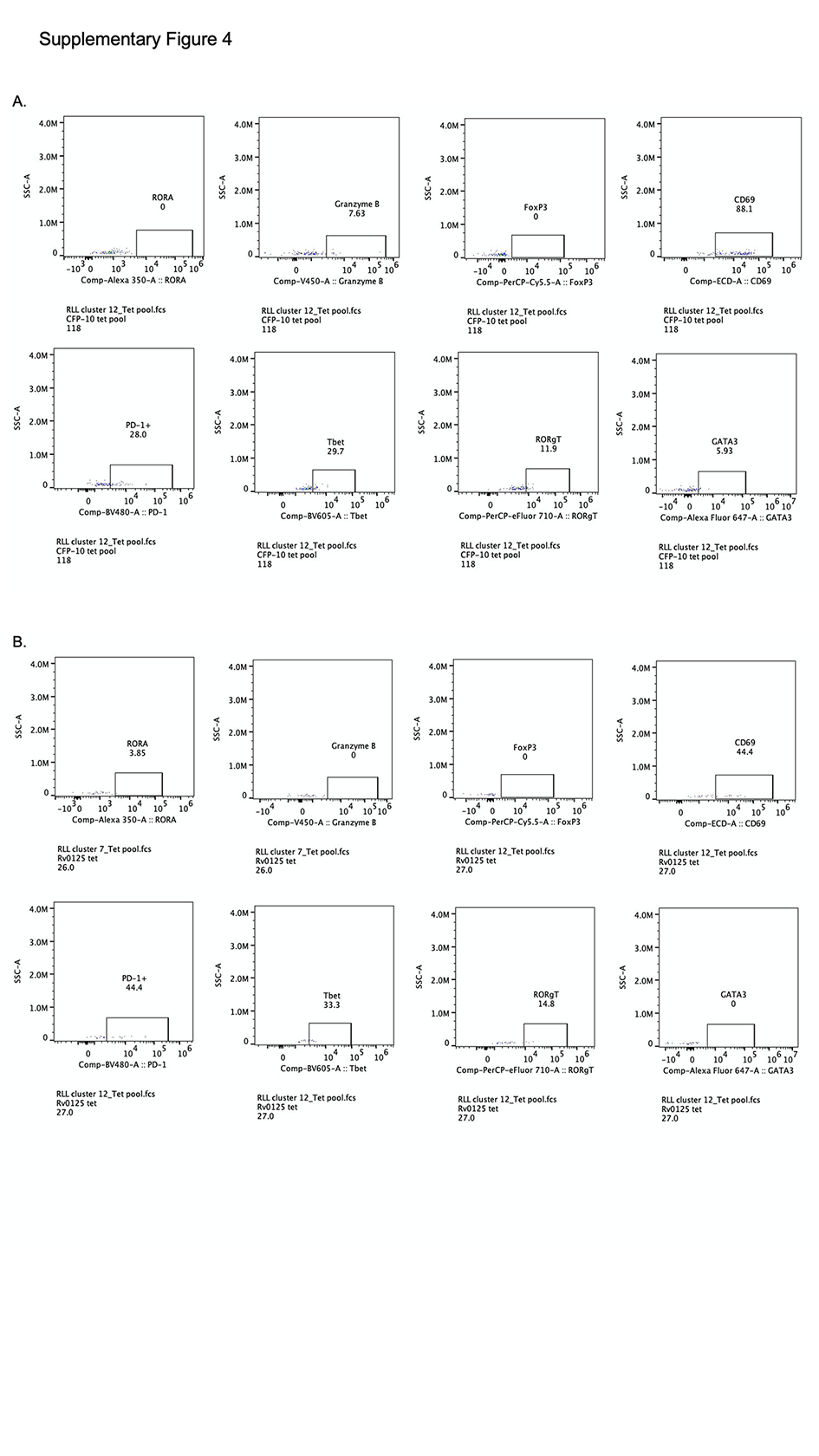

Supplement: FIG S4 [file mbio.00477-23-s0004.tif]

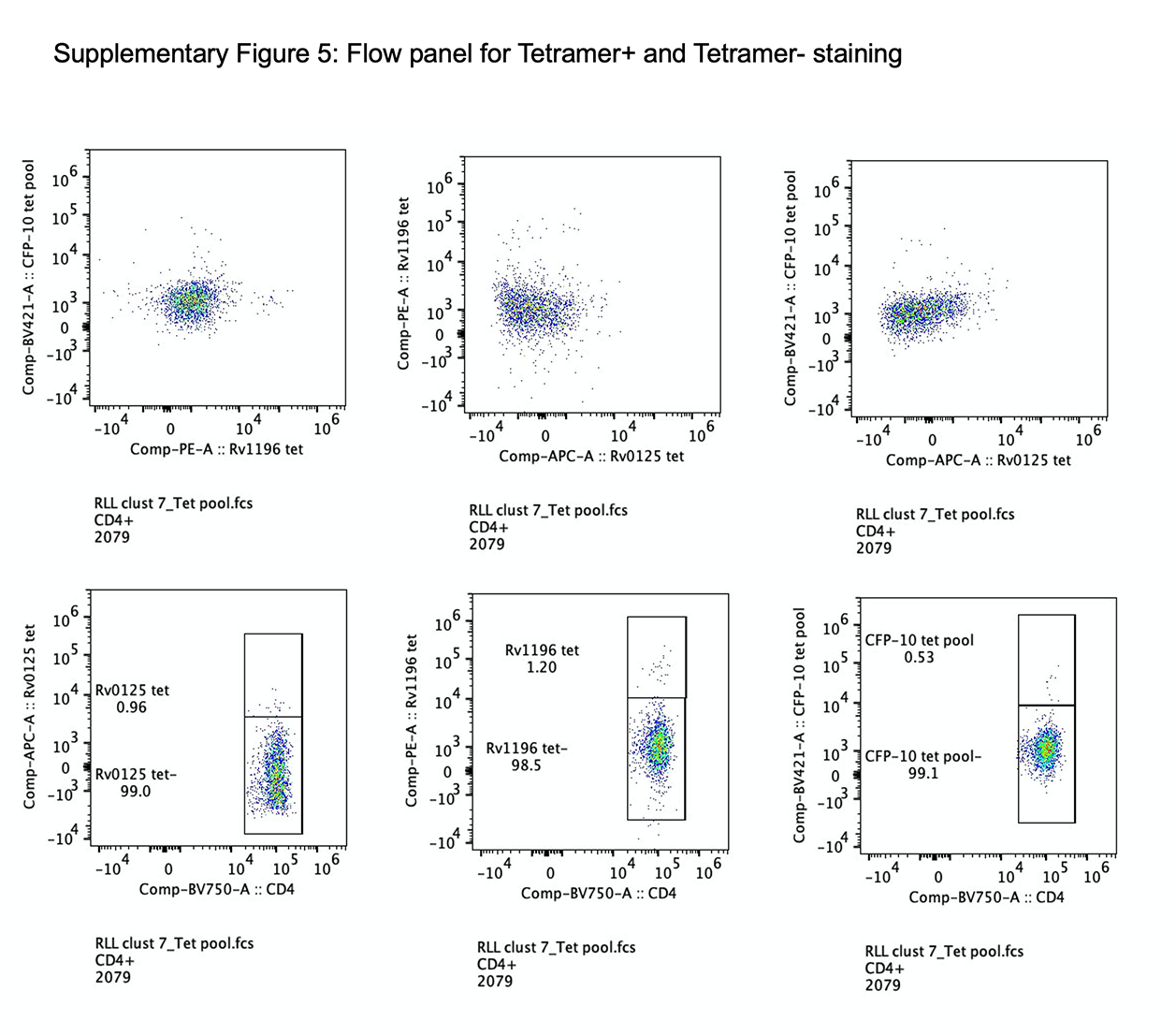

Supplement: FIG S5 [file mbio.00477-23-s0005.tif]
